# Motivation and reward processing across sex/gender and the menstrual cycle: a biopsychosocial perspective

**DOI:** 10.1186/s13293-026-00853-5

**Published:** 2026-03-04

**Authors:** Melina Grahlow, Anne Kühnel, Kristin Kaduk, Sophie Mathis, Andreas Frick, Nils B. Kroemer, Birgit Derntl

**Affiliations:** 1 Department of Psychiatry and Psychotherapy, Tübingen Center for Mental Health (TüCMH), Medical Faculty, University of Tübingen, Calwerstraße 14, Tübingen, 72076 Germany; 2 Graduate Training Centre of Neuroscience, University of Tübingen, Tübingen, Germany; 3 German Center for Mental Health (DZPG), Partner Site Tübingen, Tübingen, Germany; 4 Section of Medical Psychology, Department of Psychiatry and Psychotherapy, Faculty of Medicine, University of Bonn, Bonn, Germany; 5 Department of Medical Sciences, Experimental Cognitive and Affective Neuroscience Lab, Uppsala University, Uppsala, Sweden; 6 Department of Psychology, Uppsala University, Uppsala, Sweden; 7 Tübingen Neuro Campus, University of Tübingen, Tübingen, Germany; 8 German Center for Diabetes Research (DZD), Neuherberg, Germany; 9 LEAD Graduate School and Research Network, University of Tübingen, Tübingen, Germany

**Keywords:** Motivation, Steroid hormones, Sex/gender characteristics, Menstrual cycle, Reward sensitivity, Estradiol

## Abstract

**Background:**

Females undergo hormonal fluctuations throughout every menstrual cycle and numerously report corresponding symptoms of negative mood or decreased motivation, indicating an increased risk for affective disorders associated with altered motivational behaviour. Understanding whether sex hormones modulate sex/gender-specific behavioural variability in motivation could inform personalised interventions.

**Methods:**

To assess whether steroid hormone fluctuations and menstrual cycle phase modulate sex/gender-specific motivation, we examined 48 naturally cycling cisgender females and 46 cisgender males, aged 18–34, who performed a physical effort task while fasted (part 1, T0) and across four weeks (part 2, T1-T4). We obtained objective (invigoration and effort maintenance) and subjective (wanting and exertion) measures of motivation in response to food and monetary rewards. Menstrual cycle phases were determined based on cycle-day counting methods alongside plasma levels of estradiol, progesterone, and testosterone. We tested whether females show higher effort maintenance, males exhibit greater reward sensitivity, and explored whether motivational behaviour differs by sex/gender, cycle phase and hormonal variation.

**Results:**

Cross-sectionally, we replicated sex/gender specific reward sensitivity and valuation: Females showed more sustained effort, especially for small rewards, while males displayed more opportunistic approaches seeking monetary rewards. Longitudinally, motivation decreased during periovulatory and luteal phases, whereas levels of endogenous hormones explained little variance in instrumental effort beyond task incentives and sex/gender associations.

**Conclusions:**

Motivational behaviour in effort-based decision-making is more related to dynamic sex/gender-related factors and menstrual cycle phases overall than to short-term steroid hormone fluctuations. Our findings emphasise the importance of integrating biological, psychosocial, and physiological factors when investigating motivation. Our research has potential implications for personalised interventions and treatment of motivational deficits.

**Supplementary Information:**

The online version contains supplementary material available at 10.1186/s13293-026-00853-5.

## Highlights

- We investigated whether sex/gender differences in motivation and reward sensitivity depend on fluctuations of sex hormones in women and men.
- Women maintained effort more consistently, especially for smaller rewards, while men showed greater increases in effort for larger or monetary rewards.
- In women, motivation decreased during the periovulatory and luteal phases of the menstrual cycle, when estradiol levels were higher.
- Levels of endogenous hormones alone were not directly linked to altered motivation.
- Findings highlight the importance of considering biological, psychological, physiological and sociocultural variables when studying motivation and may help explain sex/gender-specific vulnerability to mood and motivational disorders.

**Supplementary Information:**

The online version contains supplementary material available at 10.1186/s13293-026-00853-5.

## Abstract (plain language summary)

Women often notice that their mood and motivation can change across the menstrual cycle. These changes may be linked to normal fluctuations in hormones such as estradiol, progesterone, and testosterone, but we still know little about how these hormones affect motivation in daily life. In this study, we compared motivation between women with natural menstrual cycles and men of similar age. Participants completed a task that measured how much physical effort they were willing to put in to earn different kinds of rewards – either food or money. We also measured their hormone levels and asked them about their motivation. When looking at data from one point in time, we found clear differences between women and men. Women tended to keep up their effort more consistently, especially for smaller rewards, while men increased their effort more when larger or money rewards were available. Across the menstrual cycle, motivation decreased during the middle (periovulatory) and later (luteal) phases, when estradiol levels were higher. However, hormone levels themselves were not directly linked to lower motivation. These results suggest that menstrual cycle phases can relate to how motivated people feel and behave and that this may lead to differences in motivation between women and men. Understanding how biological and psychological factors shape motivation could help explain why some people are more vulnerable to mood or motivation problems and could lead to more personalised approaches when it comes to improving mental health.

**Supplementary Information:**

The online version contains supplementary material available at 10.1186/s13293-026-00853-5.

## Background

Where there is a will, there is a way: Motivation, the driving force behind goal-directed decisions and actions, varies considerably between individuals. Cost–benefit decision-making [1] can be operationalised by assessing willingness to expend effort for a reward [2], as it reflects an integration of expected benefits and perceived costs [3–8]. Here, reports of sex/gender differences suggest women and men employ distinct approaches to achieving different goals [9, 10]: Men try to maximise gains and take more risks [11–13], while women prefer safer options [12–14] and more frequent, smaller rewards [12, 13]. In effort-based reward tasks, women prefer easy trials with smaller rewards whereas men favour difficult trials with higher rewards [15]. Our previous work found women sustained effort for small rewards and showed less opportunistic reward-seeking than men, who increased effort more in response to higher rewards [9].

Sex/gender differences in physiology, medicine, and behaviour [16–18] arise not solely from biological sex but also socio-cultural gender-related expectations for roles, norms, and behaviour, which may shape motivational behaviour [19–22]. The clinical relevance of sex/gender differences in motivation becomes evident with regard to mental health: mood and anxiety disorders are twice as prevalent in women, and substance use disorders are more common in men [23, 24].

Steroid hormones have been proposed as modulators for sex/gender differences in mental health and disease [25]. Females’ vulnerability for the development of affective disorders may correspond to ovarian hormone fluctuations [26], with over 40% experiencing symptoms of low mood and reduced interest or energy corresponding to hormonal changes across their menstrual cycle [26–29]. Sex hormones modulate the mesocorticolimbic dopamine system [30–33] in males and females and throughout the menstrual cycle [34]. Changes in the dopamine system are associated with aberrant decision-making processes and altered motivational behaviour observed in mood disorders [35, 36]. Several brain regions involved in the dopaminergic system express high densities for estrogen and progesterone receptors [26], as well as androgen receptors [37, 38]. Hence, systematic variation of ovarian hormones across the menstrual cycle and fluctuations of androgens in men may modulate motivation by interacting with relevant neurotransmitter systems [10, 26, 37, 38].

Gonadal steroid hormones are increasingly understood as regulators of motivational priorities, dynamically biasing individuals toward different classes of goal-directed behaviour. Previous research has found opposing associations of estradiol and progesterone with core motivational domains across the menstrual cycle, with estradiol being associated with approach-related behaviour and increased reward-seeking and progesterone with energy conservation. For instance, in naturally cycling women, increases in estradiol (within-cycle fluctuations measured in saliva samples) predict heightened sexual motivation and reduced food intake, whereas progesterone is associated with the opposite pattern [39, 40]. This is consistent with endocrine regulation of trade-offs between competing motivational systems. Additionally, in a review on hormonal correlates of dominance motivation, testosterone in men has been shown to facilitate approach behaviour in contexts involving competition and reward, whereas in women, the corresponding hormonal correlate seems to be estradiol [41]. At the same time, recent longitudinal research investigating naturally cycling women, women using hormonal contraception, and men largely did not replicate these previously reported hormone-motive associations and highlights variability [42].

When it comes to cost–benefit decision-making, rising levels of estradiol have been shown to positively modulate effort expenditure and enhance reward sensitivity in decision-making [43, 44]. However, findings are inconsistent in women with some studies not reporting an increase in motivation for higher rewards with rising estradiol levels [43] and others showing less sensitivity to immediate rewards with rising estradiol levels from the menstrual to the follicular phase [45]. A similar picture arises for progesterone, with hints that higher progesterone levels may positively modulate the readiness to exert effort, however, findings are, again, not extensive [10]. Regarding testosterone, a positive association between reward sensitivity and testosterone has been postulated [46, 47] and elevated testosterone levels have been proposed to lead to more risk-taking in both women and men when it comes to motivational behaviour.

Taken together, emerging evidence suggests a modulatory role of endogenous estradiol, progesterone, and testosterone in cost–benefit decision-making, indicating increased effort expenditure and reward sensitivity during high estradiol phases [10, 34, 43, 44] and elevated levels of testosterone [46–48]. Nonetheless, the nature of sex/gender-specific behavioural variability in instrumental physical effort remains unclear as findings are limited, inconclusive [10, 43], and sometimes contradictory [45].

This study aimed to 1) replicate and extend findings on sex/gender differences in physical effort allocation [9] using a cross-sectional design examining sex and gender differences and exploring associations of menstrual cycle phases and sex hormones with reward sensitivity; and 2) investigate how sex hormone fluctuations modulate reward sensitivity using a novel longitudinal, dense-sampling approach, examining within-person variations over time and across the menstrual cycle. Based on prior research [9], we hypothesised that women show higher effort maintenance, particularly for small rewards, than men, who increase effort more for larger rewards (i.e., higher reward sensitivity), resulting in similar performances in high-reward conditions. We expected women to report greater wanting and exertion, especially for small rewards. We explored whether sex/gender differences in motivation are correlated with hormonal fluctuations, both cross-sectionally and longitudinally across one menstrual cycle. Despite evidence linking higher estradiol levels to increased reward sensitivity, findings remain inconsistent, particularly with respect to within-cycle changes in effort-related decision-making. Therefore, in a preregistered analysis, we explored whether within-person changes in estradiol levels across the menstrual cycle – particularly the transition from the follicular phase to the perimenstrual phase – are associated with effort expenditure, assuming that higher estradiol levels would be correlated with higher effort expenditure [43, 44]. Similarly, we hypothesised higher reward sensitivity with elevated levels of testosterone [46, 47] but did not specify hypotheses regarding progesterone levels given inconsistent previous findings [10, 42].

## Methods

This study was approved by the ethics committee of the Medical Faculty of the Eberhard Karls Universität Tübingen (585/2020B0). We preregistered our study protocol at the Open Science Framework (https://osf.io/gpr52). The reported data are part of a larger project examining the potential link between metabolic states and dopamine-related reward learning, and the analyses we report here were only partly preregistered. We deviated from our preregistered hypothesis regarding operationalisation of the ‘premenstrual phase’ and report data from a ‘perimenstrual’ window, which is more consistent with recommended practice [49]. Participants provided written informed consent and received monetary compensation or course credit and performance-based rewards. Recruitment occurred via university mailing lists, flyers, and social media (e.g., Instagram). The sample size was selected a priori to provide at least a power of 1-β = 0.94 to study small-to-moderately sized within-subject effects (*d*_*z*_  ~  0.40) in the longitudinal analyses, corresponding to a lower-bound estimate of 80 participants after quality control. In the present study, within-subject analyses of menstrual cycle phases in women (n = 48) are powered to detect small-to-moderately sized within-subject effects (*d*_*z*_ ~ 0.43 for 1-β = 0.80), whereas cross-sectional between-subject comparisons with n = 48 women and n = 46 men are sufficiently powered to detect effects in the moderate range (*d* ~ 0.60 for 1-β = 0.80). Data were collected at the Department for Psychiatry and Psychotherapy, University Hospital Tübingen, from 04/2022–05/2024. The complete data used for the analyses, comprising trial-based behavioural measures as well as hormonal measurements, are publicly available: https://osf.io/5kbwz.

### Sample

Ninety-four cisgender participants completed all five sessions: 48 women aged 19–34 years with a Body Mass Index (BMI) between 18.8 and 33.2 kg/m^2^ and 46 men aged 18–32 with a BMI between 18.0 and 36.7 kg/m^2^. To avoid differences in hormonal fluctuations due to the use of hormonal contraceptives, only naturally cycling women were included. For sociodemographic data, see Table 1 and Supplement S1.

**Table 1:** Sociodemographic data of the study sample (*N* = 94)

	Women (n = 48)		Men (n = 46)		*t*	*p*
	*M*	*SD*	*M*	*SD*		
Years of age	23.69	3.45	24.65	3.54	-1.34	0.185
BMI (kg/m^2^)	24.43	3.90	24.95	4.24	-0.62	0.539
BDI-II	9.19	8.36	5.78	5.77	2.29	**0.025**
GERAS Femininity	4.96	0.62	4.48	0.52	3.99	** < 0.001**
Personality	5.18	0.74	4.85	0.52	2.54	**0.013**
Cognition	5.05	0.78	5.04	0.80	0.06	0.950
Activities & Interests	4.65	0.96	3.56	0.93	5.52	** < 0.001**
GERAS Masculinity	4.04	0.56	4.66	0.59	-5.14	** < 0.001**
Personality	3.96	0.65	4.36	0.72	-2.83	**0.006**
Cognition	4.47	0.92	5.08	1.09	-2.89	**0.005**
Activities & Interests	3.69	1.02	4.53	0.90	-4.20	** < 0.001**
	**n**	**%**	**n**	**%**	**p**	
Income (monthly, €)					0.361	
< 250	7	15	5	11		
250–500	6	13	7	16		
500–1000	17	36	15	33		
1000–1500	6	13	9	20		
1500–2000	2	4	3	7		
2000–3000	3	6	2	4		
3000–4000	0	0	3	7		
No answer	6	13	1	2		

Independent samples *t*-tests were used to determine significant differences between women and men for years of age, Body Mass Index (BMI), the score in the Beck Depression Inventory II (BDI-II) [50] and total and subscale scores of the Gender-Related Attributes Survey (GERAS, Supplement G1) [51]. Fisher’s exact test was used to check for significant differences in income. Statistically significant *p*-values are presented in bold

### Experimental procedure

The study comprised five experimental sessions and is reported in two parts. Part 1 reports cross-sectional data from the initial session (T0), while part 2 includes longitudinal within-subject analyses across four consecutive sessions (T1-T4). All sessions followed standardised protocols. See Supplement S2 for additional details and variables. Gender-related assessments, analyses, results, and discussion are provided in the Supplement Gender Expression.

#### Part 1

The initial session (T0) started between 7 am and 12 pm, following an overnight fast. Firstly, participants completed state ratings (Supplement S3 and G2), then provided blood samples to measure estradiol, progesterone, and testosterone levels. They completed three tasks assessing motivational behaviour; only the Effort Allocation Task (EAT) [8] is reported here. Food and monetary rewards were distributed based on task performance partly as a standardised breakfast (80% muesli, 20% snack choice; Supplement S4). After the session, participants received an extensive battery of questionnaires assessing psychopathology (e.g., Beck Depression Inventory II, BDI-II) [50], self-reported gender, and motivational aspects (Supplement S5).

#### Part 2

**Fig. 1:**
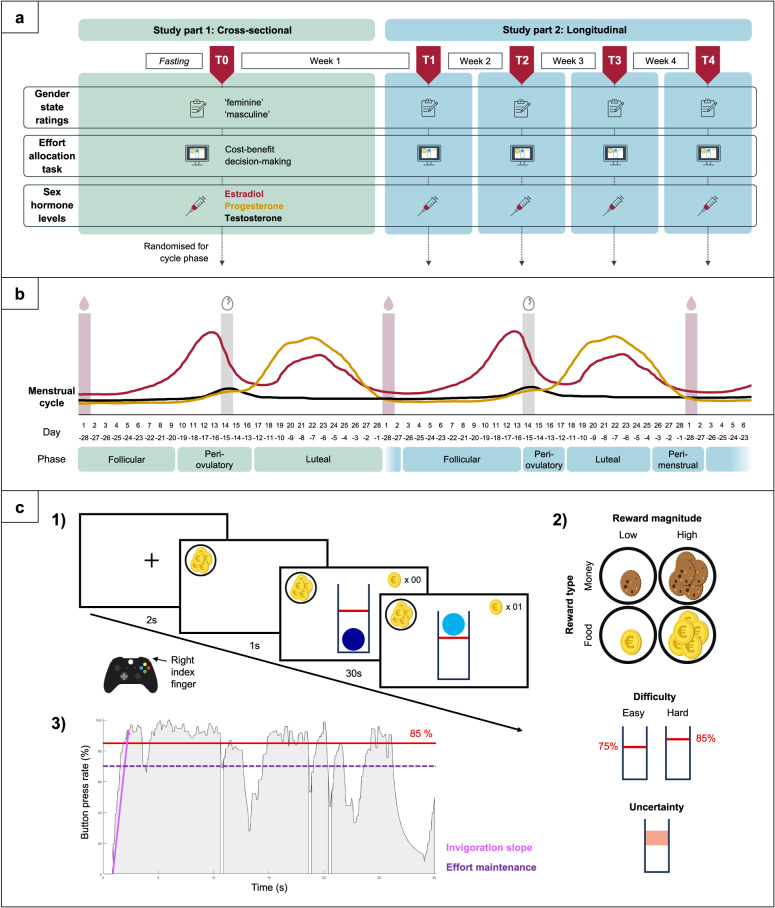
Depiction of the study design.** a** Schematic overview over the study procedure. **b** Categorisation of cycle days into phases as classified for study part 1 (T0) and study part 2 (T1-T4) with the characteristic fluctuations of sex hormones estradiol (red), progesterone (orange), and testosterone (black). Light red bars indicate menstrual onset and grey bars mark the day of ovulation in a standardised 28-day cycle. For study part 1, a follicular (cycle days + 1 to + 9), periovulatory (+ 10 to -11) and luteal group (-12 to -1) of women were determined. For part 2, the follicular phase was considered as cycle days + 3 to + 13. Days + 14 to -12 were considered the periovulatory phase. The luteal phase was estimated by reverse cycle days -11 to -4. Finally, the perimenstrual phase was estimated days -3 to + 2. **c** Schematic depiction of the effort allocation task. 1) Following a fixation cross, the reward cue is presented. To earn the depicted reward, participants repeatedly press a button with their right index finger to keep a ball above the red line. 2) As task conditions, reward magnitude (low vs. high), reward type (food vs. money), difficulty (easy vs. hard for study part 1 and alternating between different difficulty levels of 60–90% for study part 2), and uncertainty (certain vs. uncertain trials, only in study part 2) are manipulated. 3) A representative time series of a high-difficulty trial, showing effort output as button press rate, in % relative to the maximum frequency of the participants. Invigoration slopes (pink arrow) obtain how quickly participants raise their effort during a trial. Effort maintenance (dashed purple line) is assessed by the average relative frequency during the trial. Figure adapted [8] under CC BY license

Sessions T1-T4 were conducted weekly over four consecutive weeks following T0. To account for possible diurnal hormone fluctuations [52, 53], each participant was assessed at the same time across sessions. Procedures mirrored T0, including state ratings, the EAT, and blood sampling to track hormone fluctuations across the menstrual cycle (Fig. 1a). EAT rewards were paid out in snacks and monetary compensation (no muesli).

### Physiological and laboratory measures

#### Menstrual cycle

We only included women with self-reported regular menstrual cycles (mean length 21–37 days) [49]. To investigate the impact of discrete hormonal events (levels and acute changes), menstrual cycle phases were determined using calendar-based forward and backward counting [49, 54], designating the first day of menstrual bleeding as day + 1. For part 1, T0 of women were randomly distributed across cycle phases [49]: first third of their cycle (follicular group, n = 18), mid-cycle (periovulatory group, n = 12), and last third (luteal group, n = 18), with no statistically significant group differences in age or BMI. For longitudinal analyses in part 2 (T1-T4), we standardised each menstrual cycle to a length of 28 days and classified four cycle phases following recommended procedures (Fig. 1b) [49].

#### Hormone analyses

Blood samples (7.5 ml serum monovette; Sarstedt) were analysed approximately 120 min after collection at the Central Laboratory at the University Hospital Tübingen, accredited by the German Accreditation Body (DAkkS) according to DIN ISO17189. Endogenous estradiol, progesterone, and testosterone levels were measured using CE-IVD certified ADVIA Centaur immunoassays (eE2, PRGE, TSTII; Siemens Healthineers, Eschborn, Germany). Internal and external quality controls were always within the limits. For expected values [49] and adult reference intervals, see Supplement S6.

#### EAT

The EAT [8] assesses individual differences in instrumental physical effort and captures willingness to exert effort to gain rewards. To measure reward sensitivity to benefits and costs of pursuing a reward, magnitudes of reward and effort are manipulated. Participants collect food and money tokens (primary and secondary reinforcers) by exerting physical effort for high and low rewards through button pressing, analogous to lever pressing in preclinical research [55] and tokens are later exchanged for calories (muesli/snacks) or money (Fig. 1c). The prospective reward (food vs. money) was presented at the start of every trial and participants could accumulate reward points by moving and keeping a ball above a certain difficulty level (threshold indicated by a red line) by repeatedly pressing a button. Reward magnitude differed for each trial (low – 1 point vs. high – 10 points) and the difficulty of each trial was varied by alternating between 75% (easy) vs. 85% (hard) relative to participants’ individual maximum frequency in study part 1. For study part 2, difficulty levels varied between 60–90% and certain vs. uncertain trials were introduced to measure the subjective value of a reward more directly. In uncertain trials, the true difficulty was not revealed – instead of a red line, an area was shown that contained the true difficulty level but ranged from 60–90%. However, manipulations of difficulty and uncertainty were not central to the current research questions. We presented the EAT using Psychophysics toolbox version 3 [56] in MATLAB 2020a. See Supplement S7 for details.

Each effort phase of a trial concluded with two Visual Analogue Scales (0–100), assessing perceived exertion and wanting of the reward at stake. Participants were informed the task was intentionally challenging and were encouraged to rest at their convenience during trials, to recover for subsequent efforts. The EAT captures two motivational facets: invigoration, i.e., the speed at which effort is initiated during each trial (associated with subjective wanting, mostly insensitive to effort costs) and effort maintenance, i.e., how durably the level of effort is sustained throughout a trial (associated with subjective wanting and exertion, highly sensitive to the costs of effort) [8, 57], see Table 2 for a conceptual overview of motivational constructs in the EAT. The term ‘reward sensitivity’ reflects the extent to which effort allocation increases with higher reward magnitude or different reward types and therefore refers to changes in invigoration or effort maintenance as a function of experimentally manipulated task variables.

**Table 2:** Conceptual overview of motivational constructs in the effort allocation task [8]

Construct	Task-based operationalisation	Measurement	Conceptual definition
Invigoration	Slope of transition between relative frequency of button presses during a rest segment and the initial plateau during the subsequent work segment	Objective, behavioural	Initial motivation: Speed and vigour with which effort is initiated, mostly insensitive to effort costs
Effort maintenance	Average frequency of button presses during a trial	Objective, behavioural	Sustained effort over time under ongoing task demands, highly sensitive to effort costs
Wanting	Rating on Visual Analogue Scale: Desire to obtain a reward	Subjective, self-report	Subjective conscious motivational valuation of the reward, related to benefits of action
Exertion	Rating on Visual Analogue Scale: Perceived effort expenditure for the reward in the last trial	Subjective, self-report	Retrospective evaluation of how much effort was expended to obtain the reward, related to perceived effort costs

### Data analysis

Statistical analyses were conducted in R (v4.3.2; R Core Team, 2023) and R studio (v2024.4.2.764), using one-sided tests to replicate previous findings and a two-tailed $\alpha \le$0.05 for all other analyses. For further data processing, the following R packages were used: tidyverse 2.0.0, dplyr 1.1.4, ggplot 2 3.5.1, lme4 1.1–35.4, lmerTest 3.1–3 and emmeans 1.10.2. EAT data were processed in MATLAB (v2019a). For full analysis details, see Supplement S8.

#### Hormone analyses

In part 1, ANOVA retrospectively validated menstrual cycle phase classifications. In part 2, hormone data were log-transformed and subject mean-centred [58]. We fitted separate mixed-effects models for each hormone to investigate associations of within-subject fluctuations across the menstrual cycle [49] or sessions. In each model, the respective hormone was included as the outcome variable, cycle phase (for women) or session (for men) as the predictor, session to control for order effects (for women) and participant ID as the random effect. ANOVA tested for diurnal variation in men’s testosterone levels.

#### EAT

Behavioural data were segmented into work and rest phases to isolate invigoration and effort maintenance [8]. Invigoration was estimated as the slope of transition between the relative frequency of button presses during rest and the initial plateau during the following work segment (see https://github.com/neuromadlab/Tasks/tree/master/Effort_Allocation_Task/Analyses/general). Effort maintenance was calculated as the average button press frequency per trial.

As the outcomes have been previously shown to be only moderately correlated [8], single-trial estimates for invigoration and effort maintenance were entered into separate mixed-effects models. In a first step, we predicted invigoration slopes and effort maintenance using the following dummy coded predictors: reward magnitude (low vs. high), reward type (food vs. money), difficulty (study part 1: easy – 75% vs. hard – 85%; study part 2: easy – 60–69% vs. medium – 72–81% vs. hard – 84–90%) and uncertainty (certain vs. uncertain). At the participant level, for main analyses, we included sex/gender (women vs. men) as a factor, and, as nuisance covariates of no interest, we further included BMI and BDI score. To account for interindividual variance, we modelled random intercepts and random slopes for reward magnitude, reward type, difficulty, and uncertainty at the participant level. One-sided tests for hypothesised sex/gender differences in effort maintenance and two-tailed tests for all other analyses were conducted.

In a second step, to explore associations of the menstrual cycle with effort allocation in women, we predicted invigoration slopes and effort maintenance by adding menstrual cycle phase (study part 1: follicular vs. periovulatory vs. luteal; study part 2: follicular vs. periovulatory vs. luteal vs. perimenstrual) as a predictor. In post-hoc sensitivity analyses, for both women and men, we included levels of estradiol, progesterone, and testosterone (log-transformed and subject mean-centred) as predictors to explore whether variability in sex hormone levels might be associated with similar patterns.

To assess associations between sex/gender and subjective ratings of wanting (related to benefits of action) and exertion (related to costs of action), we added respective model predictors. Analyses were conducted separately for part 1 (T0) and part 2 (T1-T4). In the models for T1-T4, session number served as a variable to control for potential order effects.

Effect sizes for between-subjects effects are reported using Cohen’s *d*; for within-subjects or repeated-measures effects (including interactions), Cohen’s *d*_*z*_ is reported. Effect sizes are interpreted using conventional benchmarks (small ≈ 0.2, moderate ≈ 0.5, large ≈ 0.8). For exploratory analyses, *p*-values were adjusted for multiple comparisons using the Benjamini–Hochberg procedure to control the false discovery rate (FDR, *q*-values). FDR correction was applied separately within each family of tests, defined by outcome variable (invigoration, effort maintenance, wanting, exertion) and study part (T0 vs. T1-T4) and across all tests of interest including the relevant independent variables. Post-hoc pairwise comparisons were adjusted using Tukey’s method as implemented in the R package ‘emmeans’.

## Results

For estimates of mixed-effects models, see Table 3 (part 1) and Table 4 (part 2). Here, we report main effects and significant interactions involving sex/gender, menstrual cycle phase and sex hormones with a focus on task effects concerning reward magnitude and type. For full results, including additional effects, see Supplement S9-S10 and G4.

**Table 3:** Estimates of mixed-effects models for the Effort Allocation Task (study part 1: T0, between subjects)

	**Objective measures**								**Subjective measures**							
	**Invigoration**				**Effort maintenance**				**Wanting**				**Exertion**			
	*b*	*SE*	*t*	*p*	*b*	*SE*	*t*	*p*	*b*	*SE*	*t*	*p*	*b*	*SE*	*t*	*p*
Intercept	48.11	7.88	6.11	** < 0.001**	46.86	7.11	6.59	** < 0.001**	57.19	6.73	8.49	** < 0.001**	54.33	7.79	6.97	** < 0.001**
Reward magnitude	6.52	2.51	2.60	**0.011**	14.97	2.56	5.84	** < 0.001**	26.13	3.10	8.42	** < 0.001**	23.10	3.14	7.35	** < 0.001**
Reward type	5.34	2.85	1.87	0.065	3.27	1.90	1.72	0.088	6.76	2.79	2.42	**0.017**	5.76	2.46	2.34	**0.022**
Difficulty	2.64	2.36	1.12	0.265	-3.55	1.07	-3.33	**0.001**	-4.42	0.99	-4.46	** < 0.001**	-4.81	1.51	-3.18	**0.002**
Sex/gender	5.29	3.05	1.74	0.083	-8.52	4.51	-1.89	0.062	-11.65	4.63	-2.52	**0.014**	-12.23	4.93	-2.48	**0.015**
BMI	0.35	0.31	1.14	0.257	0.72	0.26	2.75	**0.007**	-0.09	0.24	-0.38	0.704	0.00	0.29	0.01	0.995
BDI score	-0.11	0.17	-0.64	0.526	-0.13	0.15	-0.89	0.375	-0.13	0.14	-0.97	0.334	-0.10	0.16	-0.64	0.526
Sex/gender × reward magnitude	6.50	3.58	1.82	0.072	4.62	3.66	1.27	0.209	9.47	4.43	2.14	**0.035**	10.05	4.48	2.24	**0.027**
Sex/gender × reward type	-0.10	4.07	-0.02	0.981	5.78	2.71	2.13	**0.036**	4.92	3.98	1.24	0.220	6.43	3.52	1.83	0.071
Sex/gender × difficulty	0.61	3.36	0.18	0.856	-0.74	1.51	-0.49	0.625	-0.08	1.40	-0.06	0.953	1.23	2.15	0.57	0.569
**Women**																
Intercept	39.71	11.13	3.57	** < 0.001**	31.44	11.49	2.74	**0.009**	17.11	13.41	1.28	0.208	18.57	14.33	1.30	0.202
‘Feminine’	0.17	0.13	1.30	0.202	0.37	0.14	2.69	**0.009**	0.43	0.16	2.73	**0.009**	0.40	0.17	2.36	**0.023**
‘Masculine’	0.18	0.14	1.29	0.205	0.29	0.15	1.93	0.060	0.27	0.17	1.56	0.125	0.28	0.19	1.50	0.139
‘Feminine’ × reward magnitude	-0.18	0.11	-1.74	0.087	-0.39	0.12	-3.26	**0.002**	-0.51	0.16	-3.26	**0.002**	-0.56	0.14	-3.91	** < 0.001**
‘Feminine’ × reward type	0.18	0.13	1.39	0.171	-0.05	0.06	-0.93	0.355	0.01	0.12	0.04	0.966	-0.05	0.09	-0.53	0.600
‘Feminine’ × difficulty	-0.02	0.10	-0.17	0.868	0.10	0.04	2.63	**0.012**	0.02	0.05	0.41	0.683	0.05	0.07	0.69	0.494
‘Masculine’ × reward magnitude	-0.25	0.11	-2.29	**0.025**	-0.26	0.13	-1.96	0.056	-0.35	0.17	-2.08	**0.043**	-0.39	0.16	-2.53	**0.015**
‘Masculine’ × reward type	-0.03	0.14	-0.24	0.813	-0.07	0.06	-1.19	0.240	-0.10	0.13	-0.74	0.460	-0.13	0.10	-1.26	0.213
‘Masculine’ × difficulty	-0.06	0.11	-0.61	0.545	0.08	0.04	1.98	0.054	0.07	0.05	1.40	0.169	0.04	0.08	0.50	0.622
**Men**																
Intercept	51.46	12.91	3.99	** < 0.001**	70.87	18.42	3.85	** < 0.001**	37.96	17.21	2.21	**0.032**	43.50	18.36	2.37	**0.022**
‘Feminine’	0.19	0.12	1.48	0.140	-0.17	0.19	-0.93	0.358	0.02	0.17	0.11	0.917	0.05	0.19	0.24	0.809
‘Masculine’	0.08	0.17	0.45	0.651	-0.17	0.25	-0.67	0.507	0.08	0.24	0.34	0.733	-0.04	0.25	-0.16	0.872
‘Feminine’ × reward magnitude	-0.03	0.16	-0.21	0.832	-0.03	0.14	-0.23	0.816	0.08	0.15	0.52	0.604	-0.12	0.16	-0.75	0.457
‘Feminine’ × reward type	-0.22	0.15	-1.47	0.147	0.06	0.13	0.45	0.659	-0.03	0.17	-0.18	0.858	-0.04	0.16	-0.26	0.793
‘Feminine’ × difficulty	-0.18	0.14	-1.26	0.212	0.00	0.07	-0.04	0.966	-0.01	0.06	-0.22	0.827	0.02	0.09	0.26	0.798
‘Masculine’ × reward magnitude	0.17	0.21	0.82	0.418	-0.01	0.18	-0.07	0.942	-0.07	0.20	-0.37	0.716	0.08	0.22	0.35	0.731
‘Masculine’ × reward type	-0.11	0.21	-0.51	0.610	0.25	0.18	1.36	0.182	0.35	0.22	1.55	0.129	0.32	0.21	1.48	0.146
‘Masculine’ × difficulty	0.09	0.19	0.47	0.638	-0.05	0.10	-0.53	0.600	0.07	0.08	0.81	0.423	0.01	0.12	0.04	0.967
**Women**																
Intercept	47.72	4.12	11.58	** < 0.001**	57.41	4.67	12.30	** < 0.001**	45.57	5.34	8.53	** < 0.001**	41.91	5.51	7.61	** < 0.001**
Cycle phase																
Periovulatory	13.69	6.54	2.09	**0.042**	8.44	7.37	1.15	0.258	9.92	8.43	1.18	0.245	16.81	8.69	1.94	0.059
Luteal	11.50	5.74	2.00	0.052	10.47	6.60	1.59	0.120	15.40	7.55	2.04	**0.047**	18.43	7.79	2.37	**0.022**
Cycle phase × reward magnitude																
Periovulatory	-3.28	5.20	-0.63	0.531	-4.66	6.76	-0.69	0.494	-5.61	8.65	-0.65	0.520	-8.31	8.33	-1.00	0.324
Luteal	-9.17	4.48	-2.05	**0.045**	-8.26	6.05	-1.37	0.179	-14.35	7.74	-1.85	0.070	-12.88	7.46	-1.73	0.091
Cycle phase × reward type																
Periovulatory	-4.07	6.77	-0.60	0.551	-6.02	2.79	-2.16	**0.036**	-9.83	6.14	-1.60	0.116	-5.18	4.90	-1.06	0.296
Luteal	-8.70	5.94	-1.46	0.150	-3.05	2.48	-1.23	0.226	2.85	5.49	0.52	0.606	0.83	4.37	0.19	0.851
Cycle phase × difficulty																
Periovulatory	-0.33	5.17	-0.06	0.950	-0.51	2.14	-0.24	0.812	-0.33	2.52	-0.13	0.896	-7.78	3.71	-2.10	**0.042**
Luteal	2.37	4.48	0.53	0.598	1.72	1.90	0.90	0.371	-0.91	2.22	-0.41	0.682	-2.85	3.30	-0.87	0.392
**Women**																
Intercept	24.49	21.27	1.15	0.257	43.33	23.90	1.81	0.077	63.93	27.90	2.29	**0.027**	62.15	29.50	2.11	**0.041**
Estradiol	5.31	3.95	1.34	0.187	3.79	4.46	0.85	0.400	-2.15	5.20	-0.41	0.682	-1.93	5.50	-0.35	0.728
Progesterone	0.87	2.64	0.33	0.744	-0.82	2.97	-0.28	0.784	1.35	3.47	0.39	0.699	1.17	3.67	0.32	0.751
Testosterone	2.32	8.67	0.27	0.791	-6.52	9.74	-0.67	0.507	8.74	11.37	0.77	0.446	6.45	12.02	0.54	0.594
Estradiol × reward magnitude	0.93	3.15	0.30	0.768	0.94	4.09	0.23	0.819	0.62	5.33	0.12	0.908	-0.12	5.12	-0.02	0.982
Estradiol × reward type	-2.43	4.03	-0.60	0.549	-0.72	1.70	-0.42	0.673	-1.46	3.68	-0.40	0.694	1.65	2.84	0.58	0.564
Estradiol × difficulty	1.28	3.03	0.42	0.675	0.51	1.19	0.43	0.672	0.91	1.47	0.62	0.540	1.35	2.21	0.61	0.545
Progesterone × reward magnitude	-1.28	2.09	-0.62	0.541	-0.42	2.73	-0.15	0.878	-0.23	3.55	-0.07	0.948	-0.14	3.42	-0.04	0.968
Progesterone × reward type	-2.33	2.67	-0.87	0.387	0.86	1.13	0.76	0.451	2.92	2.45	1.19	0.239	1.04	1.89	0.55	0.586
Progesterone × difficulty	-0.45	2.00	-0.23	0.822	1.50	0.79	1.89	0.066	-0.29	0.97	-0.29	0.770	0.67	1.47	0.46	0.651
Testosterone × reward magnitude	-4.84	6.75	-0.72	0.476	3.96	8.93	0.44	0.659	-2.60	11.62	-0.22	0.824	2.08	11.18	0.19	0.853
Testosterone × reward type	0.27	8.69	0.03	0.975	-4.93	3.68	-1.34	0.187	-10.76	8.02	-1.34	0.187	-9.46	6.18	-1.53	0.133
Testosterone × difficulty	1.78	6.48	0.27	0.785	-3.32	2.57	-1.29	0.204	-3.52	3.14	-1.12	0.268	-8.26	4.78	-1.73	0.092
**Men**																
Intercept	12.23	34.91	0.35	0.726	62.52	52.54	1.19	0.241	74.53	46.07	1.62	0.113	62.00	49.29	1.26	0.215
Estradiol	11.23	7.23	1.55	0.121	-2.77	11.07	-0.25	0.804	-9.46	9.69	-0.98	0.335	-12.52	10.37	-1.21	0.234
Progesterone	-7.00	5.68	-1.23	0.218	-11.31	8.77	-1.29	0.204	-17.63	7.67	-2.30	**0.027**	-14.46	8.21	-1.76	0.085
Testosterone	-0.07	7.42	-0.01	0.993	4.93	11.14	0.44	0.660	9.31	9.77	0.95	0.346	17.74	10.45	1.70	0.097
Estradiol × reward magnitude	1.83	9.08	0.20	0.841	1.79	7.91	0.23	0.822	-4.94	8.67	-0.57	0.572	-4.03	9.58	-0.42	0.676
Estradiol × reward type	-2.13	8.94	-0.24	0.813	10.45	7.93	1.32	0.195	18.58	9.78	1.90	0.064	17.14	9.31	1.84	0.073
Estradiol × difficulty	6.16	7.95	0.78	0.440	-1.27	4.21	-0.30	0.765	1.58	3.57	0.44	0.660	2.45	5.43	0.45	0.653
Progesterone × reward magnitude	9.60	7.22	1.33	0.188	4.55	6.27	0.73	0.472	8.53	6.87	1.24	0.222	6.66	7.59	0.88	0.385
Progesterone × reward type	9.42	7.12	1.32	0.192	6.30	6.29	1.00	0.322	5.74	7.75	0.74	0.464	6.17	7.38	0.84	0.408
Progesterone × difficulty	13.02	6.33	2.06	**0.042**	3.56	3.32	1.07	0.290	4.73	2.81	1.69	0.100	1.25	4.28	0.29	0.772
Testosterone × reward magnitude	-5.74	9.20	-0.62	0.535	-11.26	7.95	-1.42	0.164	-1.95	8.73	-0.22	0.824	-6.61	9.63	-0.69	0.496
Testosterone × reward type	1.74	9.06	0.19	0.849	-4.74	7.98	-0.59	0.556	-7.42	9.84	-0.75	0.455	-5.91	9.36	-0.63	0.531
Testosterone × difficulty	-10.66	8.07	-1.32	0.189	2.32	4.23	0.55	0.586	0.83	3.58	0.23	0.818	-3.59	5.45	-0.66	0.514

Analyses including all participants were run with a sample size of N = 92. Other analyses were run separately for n = 48 women and n = 46 men. Variables were coded as follows: Reward magnitude (low = 0, high = 1), reward type (food = 0, money = 1), difficulty (low = 0, high = 1) and sex/gender (female = 0, male = 1). Models for gender state ratings ‘feminine’ and ‘masculine’ were analysed for women and men separately. The model for cycle phase group (follicular = 0, periovulatory = 1, luteal = 2) was analysed for women only. Hormone measures were entered as log-transformed values in separate models for women and men. Statistically significant *p*-values are presented in bold

**Table 4:** Estimates of mixed-effects models for data of the Effort Allocation Task (study part 2: T1-T4, within subjects)

	Objective measures								Subjective measures								
	Invigoration				Effort maintenance				Wanting				Exertion				
	*b*	*SE*	*t*	*p*	*b*	*SE*	*t*	*p*	*b*	*SE*	*t*	*p*	*b*	*SE*		t	p
Intercept	57.32	11.54	4.97	** < 0.001**	70.10	6.03	11.62	** < 0.001**	59.87	7.44	8.05	** < 0.001**	65.21	8.35		7.81	** < 0.001**
Reward magnitude	5.84	1.78	3.28	**0.002**	9.72	1.86	5.22	** < 0.001**	25.52	2.61	9.79	** < 0.001**	19.41	2.55		7.62	** < 0.001**
Reward type	5.17	1.70	3.04	**0.003**	3.63	1.38	2.63	**0.009**	8.44	1.82	4.63	** < 0.001**	5.91	1.71		3.45	** < 0.001**
Difficulty																	
Medium	-2.32	1.94	-1.20	0.234	1.34	0.52	2.58	**0.011**	-0.81	0.61	-1.33	0.186	2.10	0.95		2.22	**0.029**
High	-1.47	1.79	-0.82	0.414	-0.80	0.91	-0.88	0.380	-3.07	0.90	-3.43	** < 0.001**	0.73	1.48		0.50	0.621
Uncertainty	-3.35	1.66	-2.02	**0.046**	1.51	0.61	2.45	**0.015**	-2.16	0.69	-3.14	**0.002**	3.25	1.30		2.50	**0.014**
Sex/gender	0.45	4.57	0.10	0.922	-5.65	3.84	-1.47	0.145	-9.12	4.41	-2.07	**0.041**	-9.21	4.86		-1.89	0.061
BMI	0.90	0.46	1.98	0.050	0.09	0.22	0.41	0.683	-0.06	0.28	-0.23	0.816	-0.49	0.31		-1.55	0.124
BDI score	-0.35	0.25	-1.40	0.166	-0.09	0.12	-0.69	0.490	-0.12	0.16	-0.76	0.448	-0.10	0.18		-0.56	0.579
Session	2.63	0.49	5.41	** < 0.001**	1.73	0.14	12.39	** < 0.001**	1.53	0.19	8.26	** < 0.001**	1.35	0.21		6.29	** < 0.001**
Sex/gender × reward magnitude	5.94	2.56	2.32	**0.023**	4.56	2.66	1.71	0.090	8.32	3.72	2.23	**0.028**	9.50	3.64		2.61	0.0**11**
Sex/gender × reward type	-0.90	2.45	-0.37	0.715	2.70	1.97	1.37	0.173	2.38	2.61	0.91	0.364	3.69	2.44		1.51	0.134
Sex/gender × difficulty																	
Medium	2.87	2.79	1.01	0.316	-1.24	0.75	-1.66	0.099	0.94	0.88	1.06	0.289	0.66	1.36		0.49	0.627
High	1.70	2.58	0.66	0.512	-1.29	1.30	-0.99	0.324	-0.65	1.28	-0.51	0.612	1.38	2.11		0.65	0.515
Sex/gender × uncertainty	-2.05	2.38	-0.86	0.392	-0.08	0.88	-0.10	0.924	0.84	0.98	0.86	0.395	0.81	1.85		0.44	0.664
**Women**																	
Intercept	78.39	7.29	10.76	** < 0.001**	77.87	3.32	23.49	** < 0.001**	63.33	4.33	14.63	** < 0.001**	61.06	5.24		11.66	** < 0.001**
‘Feminine’	-0.06	0.08	-0.72	0.471	-0.10	0.03	-3.53	** < 0.001**	-0.08	0.04	-1.97	**0.049**	-0.12	0.05		-2.43	**0.016**
‘Masculine’	0.07	0.08	0.86	0.390	0.01	0.03	0.53	0.593	-0.05	0.04	-1.23	0.218	-0.02	0.05		-0.50	0.620
‘Feminine’ × reward magnitude	-0.10	0.07	-1.50	0.137	0.08	0.03	2.83	**0.005**	0.12	0.04	3.08	**0.002**	0.03	0.05		0.59	0.558
‘Feminine’ × reward type	0.01	0.06	0.15	0.883	0.03	0.02	1.09	0.277	0.01	0.04	0.17	0.869	0.06	0.04		1.385	0.167
‘Feminine’ × difficulty																	
Medium	0.11	0.07	1.50	0.137	0.01	0.02	0.38	0.705	0.04	0.02	1.57	0.117	0.04	0.03		1.34	0.183
High	0.05	0.07	0.67	0.508	0.00	0.02	-0.04	0.966	-0.02	0.03	-0.49	0.624	0.01	0.04		0.17	0.863
‘Feminine’ × uncertainty	0.08	0.06	1.42	0.159	-0.02	0.02	-0.92	0.361	-0.09	0.03	-3.37	**0.001**	0.02	0.04		0.43	0.665
‘Masculine’ × reward magnitude	0.02	0.07	0.28	0.780	0.01	0.03	0.36	0.722	0.09	0.04	2.28	**0.023**	0.03	0.04		0.67	0.503
‘Masculine’ × reward type	-0.01	0.06	-0.10	0.924	-0.11	0.02	-4.64	** < 0.001**	-0.10	0.04	-2.77	**0.006**	-0.17	0.04		-4.23	** < 0.001**
‘Masculine’ × difficulty																	
Medium	-0.14	0.07	-1.94	0.055	0.04	0.02	1.96	0.053	0.04	0.02	1.53	0.127	0.09	0.03		2.57	**0.011**
High	-0.02	0.07	-0.31	0.759	0.04	0.02	1.49	0.138	− 0.01	0.03	-0.35	0.728	0.04	0.04		0.85	0.399
‘Masculine’ × uncertainty	0.01	0.06	0.15	0.878	0.02	0.02	1.00	0.321	0.02	0.03	0.82	0.413	0.05	0.04		1.31	0.191
**Men**																	
Intercept	51.32	11.88	4.32	** < 0.001**	80.64	4.87	16.55	** < 0.001**	42.63	6.09	7.00	** < 0.001**	35.04	7.44		4.71	** < 0.001**
‘Feminine’	0.01	0.11	0.12	0.905	-0.14	0.04	-3.16	**0.002**	0.10	0.06	1.67	0.096	0.20	0.07		2.77	**0.006**
‘Masculine’	0.38	0.14	2.69	**0.008**	-0.15	0.05	-3.09	**0.002**	0.07	0.06	1.01	0.311	0.07	0.08		0.81	0.420
‘Feminine’ × reward magnitude	-0.04	0.08	-0.46	0.648	0.15	0.04	3.49	** < 0.001**	-0.04	0.06	-0.67	0.502	0.13	0.06		2.01	**0.045**
‘Feminine’ × reward type	0.05	0.10	0.47	0.641	-0.05	0.04	-1.20	0.232	-0.16	0.06	-2.68	**0.008**	-0.19	0.06		-3.04	**0.003**
‘Feminine’ × difficulty																	
Medium	0.04	0.11	0.38	0.703	0.00	0.03	0.08	0.933	0.01	0.04	0.15	0.881	-0.04	0.05		-0.96	0.340
High	-0.08	0.10	-0.76	0.450	-0.03	0.04	-0.88	0.379	-0.09	0.04	-2.18	**0.032**	-0.15	0.06		-2.43	**0.016**
‘Feminine’ × uncertainty	-0.06	0.09	-0.72	0.476	-0.01	0.03	-0.21	0.832	0.03	0.03	0.83	0.410	-0.05		0.05	-1.06	0.292
‘Masculine’ × reward magnitude	-0.02	0.11	-0.18	0.857	0.14	0.05	2.86	**0.004**	0.09	0.06	1.34	0.181	0.10		0.07	1.44	0.150
‘Masculine’ × reward type	0.09	0.12	0.70	0.489	0.06	0.05	1.12	0.263	-0.05	0.07	-0.69	0.493	-0.01		0.07	-0.14	0.892
‘Masculine’ × difficulty																	
Medium	-0.09	0.13	-0.66	0.514	0.03	0.04	0.78	0.435	0.02	0.05	0.39	0.699	-0.03		0.06	-0.59	0.557
High	-0.04	0.13	-0.32	0.751	-0.04	0.04	-1.02	0.308	-0.07	0.05	-1.40	0.164	-0.03		0.07	-0.47	0.639
‘Masculine’ × uncertainty	-0.21	0.11	-1.84	0.068	0.01	0.03	0.23	0.821	-0.04	0.04	-0.86	0.392	-0.02		0.06	-0.36	0.720
**Women**																	
Intercept	77.71	3.83	20.28	** < 0.001**	72.04	2.54	28.36	** < 0.001**	59.07	3.20	18.44	** < 0.001**	54.49		3.55	15.35	** < 0.001**
Cycle phase																	
Periovulatory	-5.77	4.34	-1.33	0.184	-2.02	1.17	-1.72	0.085	-5.96	1.65	-3.62	** < 0.001**	-7.26		2.00	-3.64	** < 0.001**
Luteal	-2.97	3.85	-0.77	0.441	-2.12	1.04	-2.05	**0.041**	-2.56	1.46	-1.76	0.079	-4.66		1.77	-2.64	**0.008**
Perimenstrual	-0.35	4.45	-0.08	0.937	1.91	1.20	1.59	0.113	-2.73	1.69	-1.61	0.107	0.54		2.06	0.26	0.794
Cycle phase × reward magnitude																	
Periovulatory	-2.03	3.68	-0.55	0.581	-0.51	1.01	-0.51	0.614	2.22	1.43	1.55	0.121	0.14		1.72	0.08	0.936
Luteal	-3.80	3.26	-1.17	0.243	1.80	0.89	2.01	**0.045**	0.64	1.26	0.51	0.614	0.58		1.52	0.38	0.704
Perimenstrual	-6.70	3.68	-1.82	0.069	0.37	1.02	0.36	0.721	4.58	1.44	3.17	**0.002**	0.36		1.74	0.21	0.837
Cycle phase × reward type																	
Periovulatory	-0.66	3.64	-0.18	0.856	0.88	1.01	0.88	0.381	-0.77	1.42	-0.54	0.589	1.46		1.71	0.85	0.394
Luteal	-0.05	3.20	-0.02	0.988	2.22	0.89	2.50	**0.012**	0.99	1.25	0.79	0.431	2.54		1.51	1.69	0.092
Perimenstrual	5.42	3.62	1.50	0.134	-0.71	1.02	-0.70	0.485	-2.06	1.44	-1.43	0.153	-2.65		1.73	-1.53	0.125
Cycle phase × difficulty medium																	
Periovulatory	6.55	4.31	1.52	0.129	1.16	1.14	1.02	0.309	1.86	1.60	1.16	0.246	-0.23		1.93	-0.12	0.904
Luteal	4.61	3.75	1.23	0.219	-1.11	0.98	-1.13	0.259	1.39	1.38	1.00	0.315	-2.09		1.66	-1.26	0.208
Perimenstrual	4.66	4.28	1.09	0.277	0.08	1.12	0.07	0.943	1.17	1.58	0.75	0.456	-1.69		1.90	-0.89	0.372
Cycle phase × difficulty high																	
Periovulatory	6.53	4.54	1.44	0.151	2.58	1.24	2.08	**0.038**	4.97	1.74	2.87	**0.004**	3.17		2.09	1.52	0.130
Luteal	1.60	4.05	0.40	0.693	-0.41	1.10	-0.38	0.706	3.03	1.54	1.97	**0.049**	-0.77		1.86	-0.41	0.679
Perimenstrual	-1.28	4.53	-0.28	0.777	0.01	1.25	0.01	0.995	2.59	1.74	1.49	0.137	0.35		2.10	0.17	0.868
Cycle phase × uncertainty																	
Periovulatory	0.98	3.64	0.27	0.787	1.85	1.00	1.86	0.064	0.58	1.39	0.42	0.678	2.75		1.70	1.62	0.105
Luteal	1.50	3.19	0.47	0.638	-0.15	0.87	-0.17	0.864	0.28	1.22	0.23	0.816	0.20		1.49	0.13	0.895
Perimenstrual	2.31	3.60	0.64	0.521	-0.09	1.00	-0.09	0.929	-0.52	1.39	-0.38	0.707	-1.30		1.70	-0.76	0.446
**Women**																	
Intercept	75.67	3.24	23.35	** < 0.001**	71.42	2.46	29.01	** < 0.001**	57.02	3.11	18.34	** < 0.001**	52.01		3.44	15.13	** < 0.001**
Estradiol	-6.07	2.81	-2.17	**0.030**	-1.80	0.75	-2.39	**0.017**	-2.71	1.06	-2.56	**0.011**	-4.33		1.29	-3.37	** < 0.001**
Progesterone	-0.33	1.65	-0.20	0.839	0.03	0.45	0.06	0.953	1.00	0.63	1.60	0.110	-0.01		0.76	-0.01	0.993
Testosterone	-1.55	9.31	-0.17	0.868	2.58	2.52	1.02	0.306	5.62	3.54	1.59	0.113	1.60		4.32	0.37	0.711
Estradiol × reward magnitude	0.16	2.38	0.07	0.945	0.11	0.65	0.17	0.862	0.35	0.92	0.38	0.703	1.88		1.11	1.70	0.089
Estradiol × reward type	-0.97	2.38	-0.41	0.685	2.03	0.65	3.14	**0.002**	2.20	0.92	2.40	**0.016**	2.87		1.10	2.62	**0.009**
Estradiol × difficulty																	
Medium	0.66	2.80	0.24	0.813	-0.71	0.74	-0.96	0.339	1.22	1.04	1.17	0.241	-0.44		1.26	-0.35	0.726
High	3.32	2.96	1.12	0.262	1.03	0.80	1.29	0.196	1.51	1.12	1.35	0.178	0.38		1.35	0.28	0.779
Estradiol × uncertainty	4.07	2.37	1.72	0.086	-0.27	0.64	-0.42	0.676	-0.16	0.90	-0.17	0.863	0.73		1.09	0.67	0.501
Progesterone × reward magnitude	-0.31	1.38	-0.22	0.823	0.39	0.38	1.02	0.308	-0.13	0.54	-0.23	0.818	-0.79		0.65	-1.21	0.227
Progesterone × reward type	0.87	1.39	0.63	0.532	0.22	0.38	0.57	0.569	-0.42	0.54	-0.78	0.433	0.24		0.65	0.36	0.716
Progesterone × difficulty																	
Medium	1.64	1.62	1.01	0.311	-0.24	0.43	-0.57	0.568	-0.63	0.60	-1.05	0.295	-0.28		0.73	-0.39	0.699
High	1.32	1.74	0.76	0.448	-0.85	0.47	-1.78	0.072	0.32	0.67	0.48	0.634	-0.06		0.81	-0.07	0.942
Progesterone × uncertainty	-0.60	1.38	-0.43	0.661	0.49	0.38	1.31	0.191	-0.25	0.53	-0.47	0.636	0.06		0.64	0.09	0.925
Testosterone × reward magnitude	17.53	7.74	2.26	**0.024**	3.29	2.13	1.54	0.123	-2.25	3.00	-0.75	0.455	2.59		3.61	0.72	0.474
Testosterone × reward type	-4.71	7.75	-0.61	0.544	-4.43	2.11	-2.10	**0.036**	-2.11	2.98	-0.71	0.479	-1.17		3.58	-0.33	0.744
Testosterone × difficulty																	
Medium	2.60	9.10	0.29	0.775	-0.84	2.41	-0.35	0.727	-4.37	3.40	-1.28	0.198	0.18		4.11	0.04	0.966
High	14.34	9.68	1.48	0.139	-4.65	2.63	-1.77	0.076	-4.90	3.69	-1.33	0.184	-1.95		4.46	-0.44	0.661
Testosterone × uncertainty	-8.63	7.73	-1.12	0.264	4.27	2.09	2.04	**0.042**	0.78	2.94	0.27	0.790	1.60		3.55	0.45	0.653
**Men**																	
Intercept	78.43	2.86	27.46	** < 0.001**	66.39	2.80	23.73	** < 0.001**	49.48	3.06	16.16	** < 0.001**	44.55		3.41	13.06	** < 0.001**
Estradiol	-20.29	11.15	-1.82	0.069	4.91	3.26	1.51	0.132	-5.76	4.15	-1.39	0.166	-9.90		4.67	-2.12	**0.034**
Progesterone	-10.27	8.83	-1.16	0.245	-1.36	2.59	-0.52	0.600	-2.46	3.30	-0.74	0.457	-5.77		3.73	-1.55	0.122
Testosterone	15.63	9.26	1.69	0.092	-2.09	2.71	-0.77	0.441	-0.15	3.46	-0.04	0.966	-0.39		3.89	-0.10	0.921
Estradiol × reward magnitude	7.68	9.31	0.83	0.409	-1.91	2.78	-0.69	0.493	-1.91	3.54	-0.54	0.590	2.53		3.95	0.64	0.522
Estradiol × reward type	4.00	9.23	0.43	0.665	-1.46	2.75	-0.53	0.595	7.05	3.50	2.01	**0.044**	2.50		3.90	0.64	0.521
Estradiol × difficulty																	
Medium	23.02	10.84	2.12	**0.034**	-4.76	3.15	-1.51	0.131	1.36	4.02	0.34	0.736	3.42		4.49	0.76	0.447
High	16.10	11.37	1.42	0.157	-2.81	3.35	-0.84	0.402	4.10	4.26	0.96	0.337	3.54		4.80	0.74	0.461
Estradiol × uncertainty	2.83	9.16	0.31	0.758	-0.57	2.69	-0.21	0.832	3.48	3.42	1.02	0.309	0.76		3.85	0.20	0.844
Progesterone × reward magnitude	7.60	7.59	1.00	0.317	-1.66	2.28	-0.73	0.467	5.31	2.91	1.82	0.068	1.38		3.24	0.43	0.671
Progesterone × reward type	-1.45	7.58	-0.19	0.848	3.32	2.27	1.46	0.143	1.74	2.89	0.60	0.547	3.59		3.22	1.12	0.265
Progesterone × difficulty																	
Medium	-3.60	8.87	-0.41	0.685	0.15	2.58	0.06	0.953	2.23	3.29	0.68	0.499	1.62		3.68	0.44	0.660
High	0.19	9.46	0.02	0.984	0.83	2.80	0.30	0.765	6.38	3.56	1.79	0.074	3.87		4.03	0.96	0.336
Progesterone × uncertainty	8.38	7.52	1.11	0.266	1.66	2.22	0.75	0.454	-0.63	2.81	-0.22	0.824	1.85		3.18	0.58	0.562
Testosterone × reward magnitude	6.24	7.91	0.79	0.430	2.55	2.36	1.08	0.279	2.87	3.00	0.96	0.339	-4.10		3.35	-1.22	0.221
Testosterone × reward type	1.04	7.90	0.13	0.895	3.96	2.36	1.68	0.093	-0.67	3.00	-0.22	0.823	0.89		3.35	0.26	0.792
Testosterone × difficulty																	
Medium	-18.93	9.13	-2.07	**0.038**	-0.37	2.66	-0.14	0.890	2.30	3.39	0.68	0.497	1.73		3.79	0.46	0.648
High	-10.73	9.91	-1.08	0.279	-0.50	2.91	-0.17	0.865	0.03	3.71	0.01	0.993	3.84		4.17	0.92	0.357
Testosterone × uncertainty	-11.01	7.84	-1.40	0.161	0.07	2.31	0.03	0.974	-1.73	2.92	-0.59	0.554	1.13		3.30	0.34	0.731

Analyses including all participants were run with a sample size of N = 92. Other analyses were run separately for n = 48 women and n = 46 men. Variables were coded as follows: Reward magnitude (low = 0, high = 1), reward type (food = 0, money = 1), difficulty (low = 0, medium = 1, high = 2), uncertainty (certain = 0, uncertain = 1) and sex/gender (female = 0, male = 1). Session was used to control for order effects in all models. Models for gender state ratings ‘feminine’ and ‘masculine’ were analysed for women and men separately. The model for cycle phase (follicular = 0, periovulatory = 1, luteal = 2, perimenstrual = 3) was analysed for women only. Hormone measures were entered as log-transformed and subject mean-centred values in separate models for women and men. Statistically significant *p*-values are presented in bold

### Sex/Gender associations

#### Part 1

We replicated previous findings showing women had higher effort maintenance than men for small food rewards (*b* = -8.52, *p*_*one-tailed*_ = 0.031, *d* = -0.35, 95%CI = [-0.72,0.02]), indicating a small to moderate effect. While we did not observe a significant interaction of sex/gender × reward magnitude for effort maintenance (*b* = 4.62, *p*_*one-tailed*_ = 0.105), men increased effort for monetary rewards more than women, resulting in comparable performance in the monetary reward condition (*b* = 5.78, *p* = 0.036, *d*_*z*_ = 0.34), corresponding to a small to moderate effect. We found no sex/gender differences in invigoration (*b* = 5.29, *p* = 0.083).

**Fig. 2:**
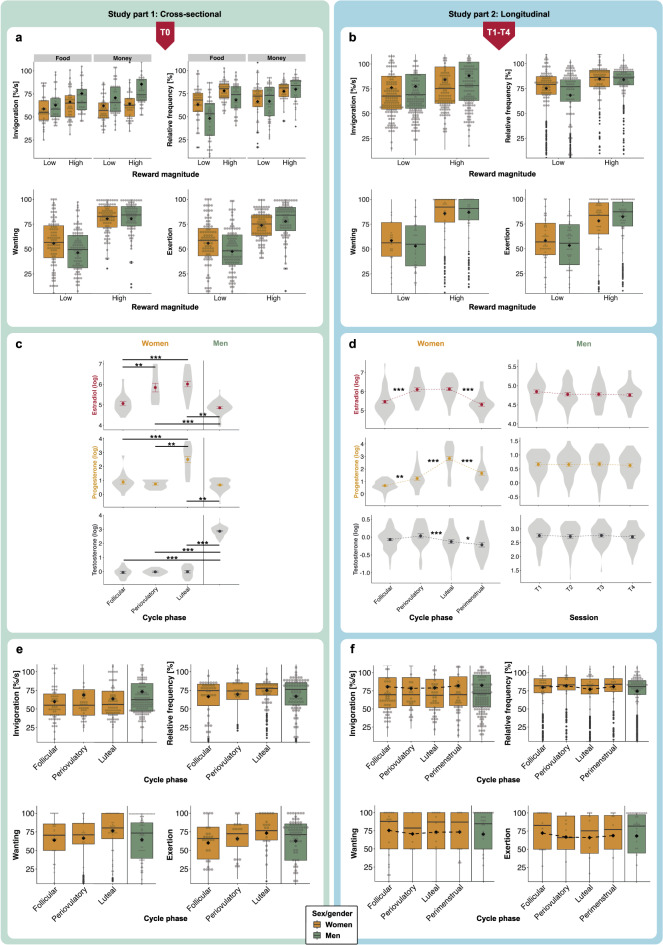
Results of cross-sectional and longitudinal trial-based data. Data from women are depicted in orange and from men in green. Results are shown separately for cross-sectional study part 1 (left panels) and longitudinal study part two (right panels). **a** Box plots showing cross-sectional data on objective (top row) and subjective (bottom row) motivation to work for low and high rewards: Women and men did not differ significantly in invigoration but in effort maintenance; women had higher effort maintenance than men for low food rewards. Men were more sensitive concerning reward type and significantly increased effort maintenance for monetary rewards. Women wanted rewards more than men and reported exerting more effort, but men showed significantly increased wanting and exertion for high rewards. **b** Box plots showing longitudinal data on objective (top row) and subjective (bottom row) motivation to work for low and high rewards: Women and men did not significantly differ in invigoration or effort maintenance. Longitudinal sex/gender differences in motivation were not robust after correction for multiple comparisons. **c** Violin plots with error bars (SE) depicting hormone levels (log-transformed) at T0 for estradiol (red), progesterone (orange) and testosterone (grey) for menstrual cycle phase groups in women and overall for men: Estradiol levels differed significantly between follicular and periovulatory, and follicular and luteal groups, but not between periovulatory and luteal. Progesterone levels differed between follicular and luteal, and periovulatory and luteal groups, but not between follicular and periovulatory. **d** Violin plots with error bars (SE) depicting hormone levels (log-transformed) across T1-T4 for estradiol (red), progesterone (orange) and testosterone (grey) in the course of the menstrual cycle for women and across sessions for men: Estradiol rose significantly from follicular to periovulatory phase, did not differ between periovulatory and luteal phase and dropped from luteal to the perimenstrual phase. Progesterone rose significantly from follicular to periovulatory phase, again from periovulatory to luteal phase and dropped from luteal to perimenstrual phase. Testosterone levels did not differ between follicular and periovulatory phase, decreased significantly from periovulatory to luteal phase, and again from luteal to perimenstrual phase. **e** Box plots showing cross-sectional motivational data for menstrual cycle phase groups in women and overall for men: Women in the periovulatory group had higher invigoration than those in the follicular group and women in the luteal group reported significantly higher wanting and exertion than those in the follicular group. **f** Box plots showing longitudinal motivational data across the menstrual cycle for women and overall for men: When in the periovulatory phase, women’s wanting and exertion ratings for rewards were significantly decreased. In the luteal phase, women significantly decreased effort maintenance and exertion ratings. *: *p* < .05, **: *p* < .01, ***: *p* < .001

Subjectively, as expected, women reported higher wanting (*b* = -11.65, *p*_*one-tailed*_ = 0.007, *d* = -0.48, 95%CI = [-0.84,-0.03]) and exertion (*b* = -12.23, *p*_*one-tailed*_ = 0.008, *d* = -0.50, 95%CI = [-1.01,-0.17]) for small rewards than men, corresponding to moderately sized effects. Men showed stronger increases in wanting (*b* = 9.47, *p*_*one-tailed*_ = 0.018, *d*_*z*_ = 0.39, 95%CI = [0.03,0.85]) and exertion (*b* = 10.05, *p*_*one-tailed*_ = 0.014, *d*_*z*_ = 0.41, 95%CI = [0.12,0.95]) for high rewards, reflecting small to moderate effects (Fig. 2a).

#### Part 2

In the following longitudinal assessment, no main effects of sex/gender were observed for invigoration (*b* = 0.45, *p* = 0.922) or effort maintenance (*b* = -5.65, *p* = 0.145). A nominal sex/gender × reward magnitude interaction indicated that men showed a stronger increase in invigoration for high rewards than women (*b* = 5.94, *p* = 0.023), however, this effect did not survive correction for multiple testing (*q* = 0.138).

Mirroring cross-sectional results, women reported nominally higher subjective levels of wanting for small rewards (*b* =  -9.12, *p* = 0.041, *q* = 0.123), while men showed greater increases in wanting (*b* = 8.32, *p* = 0.028, *q* = 0.123) and exertion (*b* = 9.50, *p* = 0.011, *q* = 0.066) in response to high rewards (Fig. 2b). Again, none of these effects remained significant after correction for multiple comparisons (all *q* > 0.05). Taken together, longitudinal sex/gender differences in motivational measures were not robust after correction for multiple testing.

### Menstrual cycle phase and hormones

#### Part 1

In women, the three groups differed significantly in estradiol, *F*(2,45) = 13.12, *p* < 0.001, and progesterone, *F*(2,45) = 30.20, *p* < 0.001, but not testosterone levels, *F*(2,45) = 0.64, *p* = 0.530 (Fig. 2c, Supplement S11), consistent with expected patterns [49] (Supplement S6).

Periovulatory women showed higher invigoration than follicular women (*b* = 13.69, *p* = 0.042, *d* = 0.39), indicating a small to moderate effect, but were less responsive to monetary incentives (*b* = -6.02, *p* = 0.036, *d*_*z*_ = -0.18, small effect). Luteal women demonstrated lower initial motivation for high incentives compared to the follicular group (*b* = -9.17, *p* = 0.045, *d* = -0.38, small to moderate effect).

Subjectively, luteal women reported higher wanting (*b* = 15.40, *p* = 0.047, *d* = 0.64) and exertion (*b* = 18.43, *p* = 0.022, *d* = 0.77) than the follicular group, reflecting moderate effects (Fig. 2e).

Behavioural effects were not explained by hormone levels in women (all *p* > 0.05), suggesting that menstrual phase rather than absolute hormone concentrations drove the effects. In men, a nominal negative association between progesterone levels and subjective wanting was observed (*b* = -17.63, *p* = 0.027), however, this effect did not survive correction for multiple testing (*q* = 0.108).

#### Part 2

In women, hormonal fluctuations across cycle phases (T1-T4) followed expected patterns [49] (Fig. 2d, Supplement S6). For men, no significant variation in testosterone levels was found across sessions or time of day (all *p* > 0.05, Supplement S11-12).

Women showed lower effort maintenance in the luteal vs. follicular phase (*b* = -2.12, *p* = 0.041, *d* = -0.11, small effect), though strong incentives such as high rewards (*b* = 1.80, *p* = 0.045, *d*_*z*_ = 0.17) or monetary rewards (*b* = 2.22, *p* = 0.012, *d*_*z*_ = 0.21, small interaction effect) attenuated this effect.

Subjectively, women also reported reduced exertion in the luteal compared to the follicular phase (*b* = -4.66, *p* = 0.008, *d* = -0.16), reflecting a small effect. Additionally, during the periovulatory phase, women indicated diminished desire (wanting, *b* = -5.96, *p* < 0.001, *d* = -0.23) and self-reported effort exertion (*b* = -7.26, *p* < 0.001, *d* = -0.25) than in the follicular phase, corresponding to small effects. During the perimenstrual vs. follicular phase, women’s wanting ratings were more sensitive to reward magnitude, with elevated wanting for high rewards (*b* = 4.58, *p* = 0.002, *d*_*z*_ = 0.31, small to moderate interaction effect), suggesting larger rewards may partially compensate for phase-related motivational declines (Fig. 2f, Supplement S13).

In analyses of continuous hormone levels, most associations in women did not survive correction for multiple testing. Although higher estradiol levels were nominally associated with reduced invigoration, effort maintenance and wanting (all *p* < 0.05, Supplement S14a), these effects did not remain significant after FDR correction (all *q* > 0.144). However, the association between higher estradiol levels and reduced self-reported exertion remained significant after correction (*q* = 0.018, *d*_*z*_ = -0.15), indicating a small effect. Nominal interactions indicated that monetary rewards attenuated the association between estradiol and effort maintenance (*b* = 2.03, *p* = 0.002, *d*_*z*_ = 0.19, *q* = 0.036, corresponding to a small interaction effect), wanting (*b* = 2.20, *p* = 0.016, *q* = 0.144) and exertion (*b* = 2.87, *p* = 0.009, *q* = 0.081), however only the interaction for effort maintenance survived FDR correction. In men, higher estradiol levels were nominally associated with reduced self-reported exertion (*b* = -9.90, *p* = 0.034), but this association did not survive correction for multiple testing (*q* = 0.612). A nominal interaction suggested that monetary incentives attenuated this association for wanting (*b* = 7.05, *p* = 0.044), which likewise did not survive FDR correction (*q* = 0.444).

Progesterone showed no significant association with motivational measures in either women or men (all *p* > 0.05). In women, testosterone showed nominal interactions with reward type, enhancing invigoration for high vs. low rewards (*b* = 17.53, *p* = 0.024, *q* = 0.270) while reducing this association for money vs. food rewards (*b* = -4.43, *p* = 0.036, *q* = 0.189, Supplement S14b), however, neither interaction remained significant after correction for multiple testing.

## Discussion

This study aimed to replicate and extend findings indicating sex/gender differences in instrumental physical effort expenditure [9]. To investigate sex- and gender-specific behavioural variability, we used a biopsychosocial approach and longitudinally examined associations of hormone fluctuations and menstrual cycle phase with objective and subjective motivational measures.

In study part 1 (cross-sectional), we replicated significant sex/gender differences in effort allocation [9]: women showed higher effort maintenance, i.e., a deliberate decision to exert effort, than men, with no sex/gender differences in invigoration, which refers to an automatic process related to motivational drive. Sex/gender-specific behaviour emerged in response to reward type: Men increased effort for monetary rewards more than women. This suggests women exerted consistent effort independent of reward characteristics, whereas men employed a more opportunistic, reward-sensitive approach, with increased motivational drive for high incentives. Our cross-sectional results align with previous literature, where women prefer frequent, smaller rewards and men pursue larger, less consistent rewards [13].

However, in study part 2 (longitudinal), we did not observe robust sex/gender differences for effort maintenance or invigoration [2, 59]. Discrepancies in findings between study part 1 and 2 might partly be explained by application mode (fasted [9] vs. arbitrary bodily states), repeated task performance and additional task conditions such as task difficulty and (un-)certain trials not detailed in this manuscript.

Subjective measures mirrored objective sex/gender differences: Cross-sectionally, women reported higher wanting and exertion, especially for small rewards, whereas ratings for large rewards were comparable between women and men. However, again, these sex/gender differences were not robust in the longitudinal assessment. The cross-sectional results suggest sex/gender-specific subjective reward valuation processes that inform the expected benefits of effort [5] and correlate with effort allocation. Notably, women may value both the benefits of rewards and the rewarding experience of exerting effort itself more than men, resulting in higher overall reward valuation (cf. the effort paradox) [2, 60]. Women may exert more effort, regardless of reward characteristics, to meet task demands and avoid aversive outcomes of loss [61, 62].

As a potential explanatory framework, sex/gender-specific subjective reward valuation may relate to the signalling function of effort in social settings. It has been suggested that gender expression, i.e., individuals’ perception of how much they embody traits associated with femininity or masculinity, may inform differences in motivation beyond biological sex: feminine traits may relate to sustained effort, signalling commitment, whereas masculine traits may encourage a more opportunistic approach [63, 64]. Similar results were observed in our supplementary analysis on the influence of Gender Expression (see Supplement G4 and G7). These self-perceived traits are shaped by experience, social desirability and societal expectations of how women and men should behave [20, 65, 66]. Social gender roles are internalised early in life and are reflected in individuals’ personality traits, whereby women have been described as, for instance, more conscientious and men as more risk-taking [67]. Feminine traits are typically associated with qualities such as agreeableness and empathy, whereas masculine traits are traditionally linked to assertiveness and competitiveness [68, 69]. A feminine, socially desirable approach to completing the reward task might be optimal task performance, including conscientious adherence to task instructions. A masculine strategy would be more competitive, focusing on maximising reward gains and conserving energy in trials with less important rewards to increase effort for more interesting rewards. This notion is consistent with previous sex/gender differences found in a gambling task, where men demonstrated a greater focus on long-term payoffs compared to women [46].

The discrepancies in findings from study part 1 and 2 highlight the variability in sex/gender related associations with motivational behaviour and emphasise the importance of considering gender alongside biological factors in understanding motivational behaviour. Furthermore, differential findings from cross-sectional and longitudinal approaches may point towards significant variability when considering motivational behaviour over time, or more specifically, across the menstrual cycle.

Our results show that cross-sectionally, motivation varied between women in different menstrual cycle phases: Women in the periovulatory group demonstrated higher initial motivation and lower sensitivity to effort costs, whereas women in the luteal group displayed higher sensitivity to effort costs and more willingness to work for rewards compared to the follicular group. These findings are consistent with previous evidence indicating increased motivation near ovulation and shifts in reward valuation during the luteal phase [30, 34, 39, 43].

Longitudinal results revealed phase-related variations in motivation following a certain pattern across the menstrual cycle. Phase-specific differences included lower subjective wanting and willingness to exert effort during the periovulatory phase, and higher sensitivity to effort costs and lower willingness in the luteal phase. Contrary to some previous findings [10, 34], these findings suggest reduced subjective motivation during phases characterised by higher estradiol levels. In the perimenstrual phase, characterised by low hormone levels, women reported increased reward sensitivity.

However, longitudinal hormone analyses revealed mostly nominal associations between hormone levels and motivational measures, with limited effects remaining significant after correction for multiple comparisons. In women, higher estradiol levels were associated with reduced self-reported exertion, whereas associations with behavioural motivational measures were not robust. Regarding testosterone, contrary to our hypothesis, we did not find robust evidence for a facilitatory role of testosterone in effort expenditure or for an overall increase in motivation with increasing testosterone levels [48].

Our findings align with prior work conceptualising steroid hormones as modulators of motivational priorities rather than direct drivers of effort-related behaviour [39, 41]. From a mechanistic perspective, this implies that biological variation in steroid hormones does not directly translate into proportional changes in instrumental effort. Instead, hormonal associations with motivation may emerge in a domain-specific and context-dependent manner, biasing reward valuation under certain conditions, e.g., across the menstrual cycle, rather than scaling effort expenditure per se [42].

While cross-sectional sex/gender differences in effort allocation were replicated [9], longitudinal sex/gender and hormone-related associations that survived correction for multiple comparisons were small. Although the study was well powered to detect small to moderate within-subject behavioural associations, detection of associations between hormones and behaviour may require larger samples due to biological variability and measurement noise. Consistent with previous findings, menstrual cycle phase was associated with variations in motivational behaviour [10, 34]. Effect sizes were predominantly small to moderate and comparable to those reported in prior studies applying effort-based decision-making paradigms [8], suggesting subtle but meaningful variation in motivational behaviour rather than strong determinative effects. Additional factors not modelled here, e.g., mood, metabolic state, physical discomfort or pain across the menstrual cycle, fatigue, stress, and gender-related motivational traits, may contribute to within-person variability in motivation.

Taken together, our results indicate that sex/gender-specific motivational behaviour may be more related to the incentive structure of the task and to menstrual cycle phase than to momentary variation in endogenous sex hormone levels. This study informs applied and clinical perspectives on motivational behaviour by delineating the limits and contextual nature of hormonal influences. By integrating repeated assessment of sex hormones with objective and subjective measures of motivation, the study demonstrates that short-term hormone fluctuations explain only a small proportion of variance in the investigated motivational behaviour. In doing so, it challenges biological models that attribute sex/gender differences in motivation primarily to circulating hormone levels [43, 44] and highlights the importance of individual differences.

In terms of applied settings, such as the workplace, the findings suggest that subtle variations in motivation across the menstrual cycle in women may interact with tasks that require prolonged effort. This perspective highlights the value of adaptively structuring work and performance environments to support motivation across individuals and contexts. The present findings are also relevant for mental disorders characterised by motivational impairment, such as affective disorders. While motivational alterations across the menstrual cycle in women were moderate in healthy individuals, they may act as vulnerability or modulatory factors that interact with mental symptoms, stress or environmental demands. Accordingly, intervention strategies targeting motivational processes (e.g., behavioural activation or incentive-based interventions) may benefit from considering menstrual cycle phase and broader contextual factors related to motivation.

### Perspectives and significance

Using a novel within-subjects design spanning one menstrual cycle, we examined both sex and gender differences. Our findings show that sex/gender-specific differences in motivation are only detectable cross-sectionally, while longitudinally, dynamic fluctuations of motivational behaviour are captured across the menstrual cycle in women. Although sessions were not optimally aligned with cycle phases, weekly sampling enhanced ecological validity. Future studies may adopt cycle-synchronised sampling to refine phase-specific associations. While we controlled for and collected measures of metabolic state in part 1 (overnight fast), future work should further consider the body’s energy state as a critical factor in motivated behaviour, as metabolic costs of (physical) effort and hormonal modulation of the dopamine system [70] link energy homeostasis [71] to reward sensitivity and instrumental action [8, 57, 72, 73]. Generalisability is limited by a predominantly Caucasian, highly educated sample. The ‘resource hypothesis’ [74, 75] suggests greater sex/gender differences arise in countries with better living conditions, where individuals pursue sex- and gender-specific goals more freely. Broader, more diverse sampling is essential to better understand how sex, gender, and corresponding socioeconomic factors interact in shaping motivation.

## Conclusion

Our comprehensive study highlights the complexity and dynamics of motivational behaviour, shaped by sex, gender, and the menstrual cycle. Women and men integrate benefits and costs differently: men display more opportunistic reward-seeking and women report greater subjective motivation, indicating sex/gender specific reward sensitivity and valuation. However, menstrual cycle phases and gender expression introduce variability, influencing consistency in effort and motivation. Levels of endogenous sex hormones alone have little to no effect on motivation and differences between study parts suggest that context, timing and physiological states may further shape motivational behaviour. Our findings emphasise the importance of considering context as well as psychosocial alongside biological factors. They may inform understanding of sex and gender differences in motivational contexts, e.g., work performance or therapeutic interventions, and guide improved treatments for mental disorders involving impaired reward processing, such as affective disorders.

## Supplementary Information

Additional file 1.

## Data Availability

The dataset supporting the conclusions of this article is available in the Open Science Framework repository, https://osf.io/5kbwz.
