# Supplementary material for: Motivation and reward processing across sex/gender and the menstrual cycle: a biopsychosocial perspective
Source: Biol Sex Differ. 2026 Mar 4;17:67. doi: 10.1186/s13293-026-00853-5 (PMC13063530; doi:10.1186/s13293-026-00853-5)
Supplement: Supplementary file 1 — Additional file 1. [file 13293_2026_853_MOESM1_ESM.pdf]

- Supplemental Material -

Motivation and Reward Processing Across Sex/Gender and the Menstrual Cycle: A Biopsychosocial Perspective

Melina Grahlow[1,2,3*], Anne Kühnel[4], Kristin Kaduk[1], Sophie Mathis[1], Andreas Frick[5,6], Nils B. Kroemer[1,3,4,7,8] & Birgit Derntl[1,3,7,9*]

[1] Department of Psychiatry and Psychotherapy, Tübingen Center for Mental Health (TüCMH), Medical Faculty, University of Tübingen, Tübingen, Germany
[2] Graduate Training Centre of Neuroscience, University of Tübingen, Tübingen, Germany
[3] German Center for Mental Health (DZPG), partner site Tübingen, Tübingen, Germany
[4] Section of Medical Psychology, Department of Psychiatry and Psychotherapy, Faculty of Medicine, University of Bonn, Bonn, Germany
[5] Department of Medical Sciences, Experimental Cognitive and Affective Neuroscience Lab, Uppsala University, Uppsala, Sweden
[6] Department of Psychology, Uppsala University, Uppsala, Sweden
[7] Tübingen Neuro Campus, University of Tübingen, Tübingen, Germany
[8] German Center for Diabetes Research (DZD), Neuherberg, Germany
[9] LEAD Graduate School and Research Network, University of Tübingen, Tübingen, Germany

*Corresponding authors
melina.grahlow@med.uni-tuebingen.de
birgit.derntl@med.uni-tuebingen.de
Calwerstraße 14, 72076 Tübingen, Germany

Supplement

S1. Sample Description, Inclusion Criteria and Compensation
S2. Description of Additionally Acquired Variables within the Project
S3. State Ratings and PANAS Items on Visual Analogue Scales (0-100)
S4. Choice of Muesli and Selection of Snacks
S5. Questionnaires
S6. Adult Reference Intervals for Blood Hormones
S7. Description of the Effort Allocation Task (EAT)
S8. Details on Data Analysis
S9. Detailed Report of Results from Study Part 1
S10. Detailed Report of Results from Study Part 2
S11. Sex Hormone Levels as Measured in Study Part 1 and 2
S12. Estimates of Mixed-Effects Models for Hormone Fluctuations (Study Part 2)
S13. Detailed Results of Motivational Behaviour Across the Menstrual Cycle (Figure)
S14. Associations of Sex Hormone Levels with Motivational Behaviour (Figure)

Supplement Gender Expression

G1. Description of the Gender-Related Attributes Survey (GERAS)
G2. Gender State Ratings
G3. Statistical Analyses including Gender Expression
G4. Results Section on Gender Expression
G5. Results of Gender State Ratings (Figure)
G6. Associations of Gender State Ratings and Motivational Behaviour (Figure)
G7. Discussion of Findings on Gender Expression

S1. Sample Description, Inclusion Criteria and Compensation

Participants provided information on their biological sex ('female' or 'male') and gender identity ('woman, 'man or 'other'). All participants were cisgender women and men, i.e., identified according to their biological sex assigned at birth.

The inclusion criteria for our study comprised participants aged 18-35 with a BMI ranging from 18.5 kg/m² to 35.0 kg/m², who were physically healthy and did not use any hormonal contraception, as determined by a telephone interview. All participants were German speaking (at least B2 level). Women who were pregnant or breastfeeding were excluded, as well as women with irregular hormonal cycles or premenstrual dysphoric disorder (PMDD) as defined by the DSM-5. Participants with a history of neurological, neurosurgical or cardiological diseases or treatments (according to self-report), current serious somatic diseases, pathological cardiac events in the past, serious brain injuries (lifetime history), hormonal diseases or serious substance or alcohol addiction (lifetime history) were excluded. Additionally, participants with metabolic disorders such as diabetes or uncompensated hypo- or hyperthyroidism, a history of surgeries on the stomach, duodenum, and/or jejunum, and any current or past-diagnosed mental illnesses (lifetime history for bipolar disorder and schizophrenia), were not eligible. The criteria also excluded individuals with current mood or anxiety disorders (with the exception of specific phobia), eating disorders of any kind, obsessive-compulsive disorders, somatization disorders, and trauma- and stressor-related disorders within the past 12 months. To ensure MRI compatibility, additional screening was conducted for factors such as intrauterine contraception with copper preparations, metal implants and large tattoos.

Participants received compensation of 160€ upon completing the study, which included payments for the initial (T0, 40€) and weekly sessions (T1-T4, 15€ each) and MRI sessions (T5 & T6, 60€ each), alongside performance-based wins from the reward tasks (*M* ~ 40€, with a range of 20-60€ and food/snacks), resulting in a total compensation of approximately 200€.

S2. Description of Additionally Acquired Variables within the Project

The full study comprised seven lab visits: one baseline session (T0), four subsequent weekly sessions to capture behavioural fluctuations across the menstrual cycle (T1-T4) and two MRI sessions (T5 and T6), where participants received a caloric load (milkshake) and water in a randomized cross-over design to investigate the effects of metabolic state on learning signals.

The initial session (T0) lasted about 3.5 h and started between 7 am and 12 pm (noon), according to the individual time at which each participant usually has their first meal of the day. Prior to T0, participants were asked to fast overnight (> 12h, where water, tea and coffee without milk or sugar were allowed). At T0, fasting levels of glucose, insulin and triglycerides were assessed, and further analyses of ghrelin and leptin are planned. We noted down the time of the participants' last meal and drink and measured body weight, height and waist circumference. In the beginning and at end of each session, participants completed a set of state ratings. MR compatibility was confirmed at T0 and due to the ongoing pandemic, participants answered questions regarding their last COVID-19 infection and whether any current symptoms were associated with COVID-19. We applied a continuous glucose monitor (FreeStyle Libre 3, Abbott) to the back of the upper arm for the duration of T0-T4 and participants were asked to track their food intake during these four weeks. Participants received a smartphone and completed up to 2 daily runs of a reinforcement learning game[1], together with an ecological momentary assessment of current metabolic and mental state. To increase commitment, participants received cumulative monetary and food rewards after each week in the following lab visit.

At T0, participants completed a Go/NoGo learning task that included rewards and punishments[2,3] (~ 30 min), where in this study we extended the original task by having all conditions once for food and once for monetary rewards. They also completed a reward rating and choice task (~ 15 min), where participants were presented with cues of primary rewards (food, music and erotic content) and after each cue, they rated wanting or liking on visual analogue scales. In the second part of the task (~ 15 min), participants were presented with a choice between two previously rated stimuli, and one was randomly selected for the participant to receive. During the subsequent consummatory phase of 30 s, participants were asked to rate their liking. To conclude T0, participants completed another set of state ratings and provided information on their strategies in the tasks and if there were any difficulties.

The following four sessions (T1-T4) lasted between 60 and 90 min and were scheduled once a week for four consecutive weeks following T0 (usually about 7 days apart). All weekly sessions were conducted at the same time within each participant (with a maximum of 2 h deviance but as little deviance as individually possible) and participants could choose their preferred time. The earliest session started at 7 am and the latest at 6 pm. At the end of each weekly session, participants received an additional standardised meal irrespective of their wins in the task, alternating between a pretzel with butter (about 330 kcal) and a fruit smoothie (about 140 kcal). Participants were asked to consume the standardised meal separately from other meals and within the same day as the respective weekly session. The goal was to be able to compare glucose levels following the standardised meals as measured by the continuous glucose monitor. To conclude all weekly sessions, participants gave information on whether the bygone month was an ordinary month concerning stress, sleep and menstrual cycle regularity.

Women continuously provided information about the day of each menstrual onset throughout participation in the study. We estimated the next day of menses for each woman based on the three previous dates of their menstruation. For study part 1, some women had their initial session in the first third of their individual cycle (cycle days +1 to +9 ~ follicular group, n = 18), others mid-cycle (+10 to -11 ~ periovulatory group, n = 12) or in the last third of their cycle (-12 to -1 ~ luteal group, n = 18). In study part 2, for statistical analyses of longitudinal data from T1-T4, we standardised each menstrual cycle to a length of 28 days by dividing the actual cycle day by length of the current cycle and multiplying by 28 and classified four cycle phases following recommended procedures. The follicular phase was considered as cycle days +3 to +13, characterised by low to slightly raised estradiol levels and low progesterone levels. Days +14 to -12 were considered the periovulatory phase, characterised by a strong rise and fall of estradiol and a slight increase of progesterone. The luteal phase was estimated by reverse cycle days -11 to -4 with high levels of both estradiol and progesterone but also possible abruptly falling hormone levels close to onset of the subsequent menses. Finally, the perimenstrual phase was estimated days -3 to +2 with falling and low levels of estradiol and progesterone.

S3. State Ratings and Items of the Positive and Negative Affect Schedule (PANAS)[4] on Visual Analogue Scales (0-100)

Category	English	German
State	Hungry	Hungrig
State	Thirsty	Durstig
State	Tired	Müde
State	Full	Satt
State	Masculine	Maskulin
State	Feminine	Feminin
State	Self-satisfaction	Selbstzufrieden
State	Physical condition	Körperliches Befinden
State	Mental condition	Psychisches Befinden
Positive Affect	Active	Aktiv
Negative Affect	Distressed	Bedrückt
Positive Affect	Interested	Interessiert
Positive Affect	Excited	Freudig erregt
Negative Affect	Upset	Verärgert
Positive Affect	Strong	Stark
Negative Affect	Guilty	Schuldig
Negative Affect	Scared	Verängstigt
Negative Affect	Hostile	Feindselig
Positive Affect	Inspired	Angeregt
Positive Affect	Proud	Stolz
Negative Affect	Irritable	Reizbar
Positive Affect	Enthusiastic	Begeistert
Negative Affect	Ashamed	Beschämt
Positive Affect	Alert	Hellwach
Negative Affect	Nervous	Nervös
Positive Affect	Determined	Entschlossen
Positive Affect	Attentive	Aufmerksam
Negative Affect	Jittery	Unruhig
Negative Affect	Afraid	Ängstlich

S4. Choice of Muesli and Selection of Snacks

Participants selected their preferred type of muesli out of three options (dried fruits, chocolate cookie, or honey nut; Peter Kölln GmbH & Co. KGaA, Elmshorn, Germany) or vegan alternatives. Individual breakfast servings would consist of muesli and cow milk (Weihenstephan preserved milk 3.5%; Molkerei Weihenstephan GmbH & Co. KG, Freising, Germany) or oat milk (Oatly Hafer Barista Edition, Oatly, Malmö, Sweden), depending on preference, scaled according to the food points earned during the tasks. Participants could drink water ad libitum during each session.

Participants could choose from a wide selection of snacks as rewards for their performance in the tasks. They could choose between muesli bars of CORNY nussig / Schoko (Schwartauer Werke GmbH & Co. KGaA, Bad Schwartau, Germany), Pick Up! Choco (Leibniz; Bahlsen GmbH & Co. KG, Hannover, Germany), HARIBO Goldbären mini (HARIBO GmbH & Co. KG, Bonn, Germany), an apple, Kinder Bueno mini (Ferrero; Ferrero International S.A., Luxembourg), Bahlsen Hit mini (Bahlsen GmbH & Co. KG, Hannover, Germany), Milka mini (Mondelez International Inc., Deerfield, IL, USA), Mini Manner (Manner; Josef Manner & Comp AG, Vienna, Austria), and Lindt mini (Lindt & Sprüngli AG, Kilchberg, Switzerland).

S5. Questionnaires

Subsequent to the initial session (T0), participants were provided with an extensive battery of questionnaires assessing a comprehensive range of psychological constructs, behavioural tendencies and dimensions of mental health, which they could complete at their convenience. Questionnaires were employed via an online questionnaire that was generated using SoSci Survey[5] and was made available via www.soscisurvey.de. Demographic data for the study were collected through a series of questions designed to capture key personal information. Participants were asked about their biological sex (female/male) and their gender identity (woman/man/other). Age information was gathered, as well as current country of residence. Level of education was assessed by asking for the highest level of education completed, with options ranging from still being a student to various school and university degrees. Participants were also inquired about their current occupation and their approximate monthly net income, defined as the total income after taxes and social security deductions. Options for income ranged from less than 250€ to 5000€ or more, with an option for preferring not to answer. Additionally, participants were asked to provide their current weight in kilograms and height in centimetres. Finally, medication use was assessed, and if affirmative, participants were asked to specify the medication.

Afterwards, an extensive battery of questionnaires was presented. We applied the Premenstrual Symptoms Sreening Tool (PSST)[6], Alcohol Use Disorders Identification Test (AUDIT)[7], Fagerström Test for Nicotine Dependence (FTND)[8], Cannabis Use Disorders Identification Test (CUDIT)[9], Gender-Related Attributes Survey (GERAS)[10], Rosenberg Self-Esteem Scale (RSES)[11], Beck Depression Inventory (BDI-II)[12], Trait Sexual Motivation Questionnaire (TSMQ)[13], Behavioral Inhibition/Approach System (BIS/BAS)[14], Salzburg Stress Eating Scale (SSES)[15], Food Cravings Questionnaire (FCQ-T-r)[16], Fragebogen zum Essverhalten (FEV)[17], Power of Food Scale (PFS)[18], Eating Disorder Examination Questionnaire (EDEQ)[19], Yale Food Addiction Scale (YFAS)[20], Skalen zum Erleben von Emotionen (SEES)[21], International Physical Activity Questionnaire (IPAQ)[22], Lille Apathy Rating Scale (LARS)[23], Liebowitz Social Anxiety Scale (LSAS)[24], Snaith Hamilton Pleasure Scale (SHAPS)[25] and State-Trait-Anxiety Inventory (STAI-T)[26].

S6. Adult Reference Intervals for Blood Hormones

As per the information from the Central Laboratory of the Institute of Clinical Chemistry and Pathobiochemistry at the University Hospital Tübingen, the following data was obtained on samples from apparently healthy individuals, based on the inner 95% interval and the following reference ranges were established / ND = not detectable.

Table S6. *Adult reference intervals*

	**Enhanced Estradiol**		**Progesterone**		**Testosterone II**		
	ADVIA Centaur eE2		ADVIA Centaur PRGE (ACS:180)		ADVIA Centaur TSTII		
						Central 90th reference interval	
**Sample category**	*Median* (pmol/L)	*Range* (pmol/L)	*Median* (nmol/L)	*Range* (nmol/L)	*Median* (nmol/L)	*5th* (nmol/L)	*95th* (nmol/L)
**Menstruating females**							
adults < 50 years of age (day in cycle relative to LH peak)					0.62	0.29	1.21
Follicular phase (-12 to -4 days)	190.1	71.6-529.2	1.37	ND-4.45			
Midcycle (-3 to +2 days)	562.5	234.5-1309.1					
Mid luteal phase			47.13	14.12-89.14			
Luteal phase (+4 to +12 days)	321.4	204.8-786.1	40.51	10.62-81.28			
**Males**							
adults < 50 years of age	91.1	ND-146.1	1.72	0.89-3.88	14.22	6.85	23.23

S7. Description of the Effort Allocation Task (EAT)[27]

At T0, to estimate the maximum frequency of button presses for each individual, participants completed a training before commencing the actual task. For two initial trials of 10 s length each, a tube containing a blue ball appeared on the screen. Participants could move the ball upwards within the tube by repeatedly pressing a button on an Xbox 360 controller (Microsoft Corporation, Redmond, WA) with their right index finger. A blue tangent line on the vertical axis was pushed upwards by moving the ball upwards, marking the highest position reached by the ball so far. Even when participants stopped pressing the button and the ball dropped, this line remained at the highest reached position and depicted the maximum frequency of button presses achieved so far (‘peak’). Participants were encouraged to push the line as high as they could. We used a moving average algorithm with exponential weighting (λ = .06) to smooth the movement of the ball for display on screen. Hence, when participants stopped working or reduced the frequency of button presses, the ball fell quickly yet slowed down.

At T0, T1 and T2, participants completed eight practice trials before commencing the actual task. Presented in a randomized order including a short break after half of the trials, these trials comprised all possible combinations of reward magnitude (low vs. high), reward type (food vs. money) and difficulty (easy vs. hard) of the EAT. By use of these practice trials, the maximum frequency of button presses was updated if participants exceeded the level previously achieved during training. After completing the practice trials, participants received feedback about the reward they would have won as a reference for the following experiment.

In the EAT itself, a prospective reward, which was either food (indicated by a cookie) or money (indicated by a coin), was presented for 1 s at the start of every trial. Reward magnitude differed for each trial: The magnitude of the reward at stake was varied with one symbol signalling a low reward magnitude (1 point/s) and several symbols indicating a high reward magnitude (10 points/s). Following the depiction of the prospective reward, a tube containing a blue ball was presented on the screen. By repeatedly pressing a button on an Xbox 360 controller (Microsoft Corporation, Redmond, WA) with their right index finger, participants could earn reward points by vertically moving the ball above a certain difficulty level. Difficulty was indicated by a red line that corresponded to a threshold relative to the individual maximum frequency. For every second that the ball was held above the line (indicated by a change of colour from dark to light blue), reward points were accumulated and tracked by a counter in the upper right corner of the screen. With a counterbalanced order across participants, the difficulty of each trial was varied by alternating between 75% (easy) and 85% (hard) for study part 1 (T0) and varied between different difficulty levels (60-90% with steps of 3% in between, i.e., 11 different levels) for study part 2 (T1-T4). Moreover, in some trials (introduced in T1), the true difficulty was not revealed, resulting in certain vs. uncertain trials. In uncertain trials, a red area was shown instead of the red line, that contained the true difficulty level but ranged from 60-90%, and the counter tracking rewards points was hidden. That way, participants did not know whether they were exerting the necessary effort to gain the depicted reward. After completion of a certain trial, the counter on the screen tracked the total of rewarded seconds during each trial, whereas after an uncertain trial, a tube with a red line presenting the actual threshold was shown, together with the number of points won.

At T0, the task comprised a total of 48 trials, each with a duration of 30 s, including 6 repetitions of all combinations of the conditions reward magnitude (low vs. high), reward type (food vs. money) and difficulty (easy – 75% vs. hard – 85%). For T1-T4, each run of the task consisted of 44 trials of 22 s. Again, reward magnitude (low vs. high) and reward type (food vs. money) as well as difficulty (60-90%) varied. Additionally, we included an uncertainty condition (certain vs. uncertain trials). This resulted in 88 combinations of possible trials. All trial combinations were presented twice across the four experimental sessions. Specifically, the 88 combinations were randomly split for the first two sessions (T1 and T2) and again during the second pair of sessions (T3 and T4) while ensuring that the average reward magnitude and difficulty was matched for all sessions. Participants could take two short breaks to recuperate. The EAT took about 35 min in total.

After completing the task, the total amount of tokens participants had collected was shown on the screen. Monetary and food tokens were exchanged for money or calories (muesli and snacks) at a rate of 1 kcal or 1 cent per five points at the end of each session. Only completed sessions were rewarded in tokens.

The following instructions were given to participants about the task (in German): “[Training] On the screen, you will see an open container with a blue ball inside. When you press the right-hand side button on the controller, the ball will move upward. The highest position you reach will be shown with a blue line. [Practice] Next, you will practice the actual task: In this phase, the line will no longer move together with the ball. Try to use button presses to push the ball upward and keep it above a red line. You earn points for every full second that the ball stays above that red line. In some rounds, you can earn money points. After completing the task, you will receive a payout corresponding to the number of money points you collected. In other rounds, you can earn calorie points, which will be paid out as a snack of equivalent value after the task. The following reward conditions apply [corresponding icons shown for each condition]: 1 money-point per second, 1 food-point per second, 10 money-points per second, 10 food-points per second. The reward type remains the same for one 24-second round. After each round, you will see how many points you earned. Throughout the experiment, there will be different difficulty levels. It will therefore not always be possible to keep the ball fully above the line at all times. One way of dealing

with this is to take breaks even during a round so that you can apply stronger pressure again afterward. After each round, you will be asked two questions: How much did you exert yourself in this round? How much did you want to receive the reward in this round? You can use the joystick to move the slider along a scale (from “not at all” to “very”) to answer. Confirm your answer by pressing button A. Please note that you only have a limited amount of time to answer. Therefore, try not to think too long - answer spontaneously.”

S8. Details on Data Analysis

Statistical Threshold and Software

Statistical analyses were conducted in R version 4.3.2 (R Core Team, 2023) and R studio version 2024.4.2.764, using one-sided tests for replication of previous results and a two-tailed $\alpha \leq .05$ for all other analyses. For further data processing, the following R packages were used: tidyverse 2.0.0, dplyr 1.1.4, ggplot 2 3.5.1, lme4 1.1-35.4, lmerTest 3.1-3 and emmeans 1.10.2. Data of the EAT was processed with MATLAB version 2019a.

Hormone Analyses

Distribution of hormone levels was inspected by visual checks and Shapiro-Wilk tests and all significantly deviated from normality (all $p < .001$). To achieve normal distribution, hormone levels were log-transformed for statistical analyses. To investigate influences of within-subject hormonal fluctuations across the menstrual cycle[28], subject mean-centering was performed for hormonal data from T1-T4[29].

To retrospectively validate the classified phases of the menstrual cycle, we used analyses of variance (ANOVA) to check if levels of hormones were significantly different in the three cycle phase groups (study part 1, T0). To investigate whether hormones fluctuated significantly across classified phases of the menstrual cycle for women and across experimental sessions for men (study part 2, T1-T4), we fitted mixed-effects models for estradiol, progesterone and testosterone separately. In each model, the respective hormone was included as the outcome variable, cycle phase (for women) or session (for men) as the predictor, session to control for order effects (for women) and participant ID as the random effect. For men, we checked whether testosterone levels significantly differed in participants that completed sessions T1-T4 in the morning (between 7 am and 12 pm) compared to those that took part in the afternoon (between 12 pm and 6 pm), using ANOVA.

Effort Allocation Task

We divided the behavioural data into segments of work and rest, to isolate the motivational facets invigoration and effort maintenance[27]. To analyse invigoration, we estimated the slope of the transition between the relative frequency of button presses during a rest segment and the initial plateau during the following work segment (MATLAB findpeaks function). To calculate effort maintenance, we averaged the frequency of button presses during each trial, capturing how much effort participants produce over time.

Single-trial estimates for invigoration and effort maintenance were entered into separate mixed-effects models. In a first step, we predicted invigoration slopes and effort maintenance using the following dummy coded predictors: reward magnitude (low vs. high), reward type (food vs. money), difficulty (study part 1: easy – 75% vs. hard – 85%; study part 2: easy – 60-69% vs. medium – 72-81% vs. hard – 84-90%) and uncertainty (certain vs. uncertain). At the participant level, we included sex/gender (women vs. men) as a factor, as well as BMI and BDI score. To account for interindividual variance, we modelled random intercepts and random slopes for reward magnitude, reward type, difficulty, and uncertainty at the participant level. We conducted one-sided tests for specific hypotheses regarding sex/gender differences in effort maintenance and two-tailed tests for all other analyses.

In a second step, to assess specific associations of the menstrual cycle and sex hormones with effort allocation, we predicted invigoration slopes and effort maintenance by adding the following predictors for women: menstrual cycle phase (study part 1: follicular vs. periovulatory vs. luteal; study part 2: follicular vs. periovulatory vs. luteal vs. perimenstrual) and levels of estradiol, progesterone and testosterone (log-transformed and subject mean-centred), while for men only sex hormone levels were added. Furthermore, to investigate whether gender expression might influence motivational behaviour differentially from sex/gender identity, we included 'feminine' and 'masculine' gender state ratings as predictors.

To examine associations between sex/gender and gender expression with subjective ratings of wanting (related to benefits of action) and exertion (related to costs of action), we used mixed-effects models to predict wanting or exertion as outcomes with the same predictors as stated above.

Analyses were conducted separately for study part 1 (T0) and part 2 (T1-T4). In the models for T1-T4, session number served as a variable to control for potential order effects. Effect sizes for between-subjects effects are reported using Cohen's *d*, for within-subjects or repeated-measures effects (including interactions), Cohen's d_z is reported.

S9. Detailed Report of Results from Study Part 1

Invigoration

In the initial session T0, women and men did not differ significantly in invigoration (p = .083). All participants showed increased invigoration slopes for trials with high compared to low reward magnitude (p = .011). Women in the periovulatory group had increased invigoration slopes compared to women in the follicular group (p = .042). A significant interaction of cycle phase x reward magnitude revealed that women in the luteal group had decreased invigoration slopes for high rewards compared to women in the follicular group, i.e. the effect of reward magnitude on invigoration slopes was stronger in the luteal compared to the follicular group (p = .045). There were no significant influences of hormone levels on any of the task variables for women (all p > .05). For men, there were no significant main effects of hormone levels (all p > .05), but a significant interaction of progesterone x difficulty suggests that men with higher progesterone levels have increased invigoration in difficult trials (p = .042). For women, we did not see any overall influence of 'feminine' and 'masculine' gender ratings on invigoration (all p > .05), but a significant interaction of 'masculine' x reward magnitude revealed that women who rated themselves more 'masculine', had lower invigoration for high rewards (p = .025). For men, there were no significant influences of 'feminine' or 'masculine' ratings on any of the task variables (all p > .05).

Effort Maintenance

For T0, we did not find a significant sex difference in effort maintenance overall (p = .062), but a significant sex x reward type interaction suggests that women and men adjusted their performance differentially in response to money rewards: Men increased their performance in response to money rewards significantly more than women (p = .036). All participants exerted more effort for trials with high compared to low rewards (p < .001) and for trials with low compared to high difficulty (p = .001). Participants with higher BMI exerted more effort overall (p = .007). For women, there were no significant differences in effort maintenance between cycle phase groups overall (all p > .05). A significant cycle phase x reward type interaction showed that women in the periovulatory group increased their effort for money rewards significantly less than women in the follicular group (p = .036). We found no significant influences of hormone levels on any of the task variables for women or for men (all p > .05). Women with higher 'feminine' ratings showed higher effort maintenance than women with lower 'feminine' ratings (p = .009). A significant interaction of 'feminine' x reward suggests that the increase in effort maintenance for high rewards is attenuated for women with higher 'feminine' ratings (p = .002). The interaction of 'feminine' x difficulty revealed that the decrease in effort maintenance due to high task difficulty was less pronounced for women with higher 'feminine' ratings (p = .012). For men, there were no significant influences of 'feminine' or 'masculine' ratings on any of the task variables (all p > .05).

Wanting

We found a significant sex difference in wanting, with women indicating that they wanted rewards significantly more than men (p = .014) and a significant sex x reward magnitude interaction showed that men had a stronger increase in their wanting ratings for high rewards compared to women (p = .035). All participants indicated they wanted high rewards more than low rewards (p < .001) and money more than food rewards (p = .017). They wanted rewards less when task difficulty was high (p < .001). Women in the luteal group wanted rewards more than women in the follicular group (p = .047). For women, there were no significant influences of hormone levels on any of the task variables (all p > .05), but for men, we found a significant influence of progesterone levels on wanting: The higher progesterone levels, the less wanting men indicated for the reward at stake (p = .027). Women with higher 'feminine' ratings indicated higher wanting compared to women with lower 'feminine' ratings (p = .009). Significant interactions of 'feminine' x reward magnitude and 'masculine' x reward magnitude showed that the positive effect of reward magnitude on wanting ratings was attenuated for women with higher 'feminine' ratings (p = .002) and with higher 'masculine' ratings (p = .043). For men, there were no significant influences of 'feminine' or 'masculine' ratings on any of the task variables (all p > .05).

Exertion

Analyses revealed a significant sex difference in exertion, with women indicating significantly higher exertion than men overall (p = .015) and a significant sex x reward magnitude interaction revealed that men indicated a higher increase in exertion for high rewards than women (p = .027). All participants indicated they exerted more effort for high rewards (p < .001), money rewards (p = .022) and when task difficulty was low (p = .002). Women in the luteal group indicated more exertion for rewards than women in the follicular group (p = .022). A significant cycle phase x difficulty interaction showed that women in the periovulatory group indicated less exertion for difficult trials than women in the follicular group (p = .042). Neither for women nor for men, there were any significant influences of hormone levels on any of the task variables (all p > .05). Women with higher 'feminine' ratings reported higher exertion than women with lower 'feminine' ratings (p = .023). Significant interactions of 'feminine' x reward magnitude and 'masculine' x reward magnitude suggest that women with

higher ‘feminine’ ratings ($p < .001$) and women with higher ‘masculine’ ratings ($p = .015$) indicated a reduction in exertion for high rewards compared to women with lower ‘feminine’ or ‘masculine’ ratings, respectively. For men, there were no significant influences of ‘feminine’ or ‘masculine’ ratings on any of the task variables (all $p > .05$).

S10. Detailed Report of Results from Study Part 2

Invigoration

In study part 2, women and men did not differ significantly in invigoration (p = .922). A significant sex x reward magnitude interaction showed that women and men adjust their invigoration differentially in response to high rewards: Men had a stronger increase in invigoration slopes in response to high rewards compared to women (p = .023). All participants showed increased invigoration slopes for trials with high compared to low rewards (p = .002), for money compared to food rewards (p = .003) and for certain compared to uncertain trials (p = .046). There was no significant influence of cycle phase on invigoration slopes in women (all p > .05). Invigoration slopes were decreased when women had higher levels of estradiol than when estradiol levels were lower (p = .030). A significant testosterone x reward magnitude interaction showed that invigoration slopes were increased for high rewards if testosterone levels were higher (p = .024). There were no main effects of any hormone levels on invigoration in men (all p > .05). A significant interaction of estradiol x difficulty indicated that invigoration was increased in trials where estradiol levels were high, and task difficulty was medium compared to low (p = .034). A significant testosterone x difficulty term revealed that men's invigoration slopes were decreased in trials with high testosterone levels and medium task difficulty compared to low (p = .038). For women, we did not see any influence of 'feminine' and 'masculine' gender ratings on invigoration (all p > .05). For men, invigoration slopes were increased with higher 'masculine' ratings (p = .008).

Effort Maintenance

We did not find a significant sex difference in effort maintenance (p = .145). All participants exerted more effort for trials with high compared to low reward magnitude (p < .001), for money compared to food rewards (p = .009), for trials with medium compared to low difficulty (p = .011) and for uncertain compared to certain trials (p = .015). Women exerted significantly less effort when they were in the luteal phase compared to when they were in the follicular phase (p = .041). Significant interactions of cycle phase x reward magnitude and cycle phase x reward type suggest that during the luteal phase, women increase their effort maintenance in response to high rewards (p = .045) and money rewards (p = .012) compared to the follicular phase. The interaction of cycle phase x difficulty revealed that during the periovulatory phase, women increase their effort in trials with high compared to low difficulty (p = .038). Women had significantly less effort maintenance with higher estradiol levels (p = .017) but the estradiol x reward type interaction indicated that the effect of estradiol on effort maintenance depends significantly on whether women work for money or for food (p = .002). A significant interaction of testosterone x reward type indicated a weaker influence of higher testosterone on effort maintenance for money rewards (p = .036). The significant term of testosterone x uncertainty showed a stronger effect of testosterone on effort maintenance for uncertain compared to certain trials (p = .042). We found no significant influences of hormone levels on any of the task variables for men (all p > .05). Women showed significantly lower effort maintenance with higher 'feminine' ratings (p < .001) and the significant interaction of 'feminine' x reward magnitude revealed that women with higher 'feminine' ratings increased their effort maintenance for high compared to low rewards (p = .005). A significant term of 'masculine' x reward type indicated that women with higher 'masculine' ratings showed a decrease in effort maintenance for money compared to food rewards (p < .001). For men, there were significant associations of both higher 'feminine' (p = .002) and 'masculine' (p = .002) ratings with decreased effort maintenance. Significant 'feminine' x reward magnitude and 'masculine' x reward magnitude interactions revealed that men with higher 'feminine' ratings (p < .001) and higher 'masculine' ratings (p = .004) showed an increase in effort maintenance for high compared to low rewards.

Wanting

We found a significant sex difference in wanting, with women indicating that they wanted rewards significantly more than men (p = .041). A significant sex x reward magnitude interaction showed that men showed a greater increase in wanting ratings for high rewards compared to women (p = .028). All participants indicated they wanted high rewards more than low rewards (p < .001) and money more than food rewards (p < .001). They wanted rewards less when task difficulty was high compared to when it was low (p < .001) and they wanted rewards less when the trial was in the uncertain condition compared to the certain condition (p = .002). Women wanted rewards significantly less when they were in the periovulatory compared to the luteal phase (p < .001). We found a significant interaction of cycle phase x reward magnitude, showing that women indicate higher ratings of wanting for high compared to low rewards when they are in the perimenstrual phase (p = .002). A significant interaction of cycle phase x difficulty revealed that women report greater changes in wanting ratings in response to trials with high difficulty when they are in the periovulatory (p = .004) and luteal phase (p = .049) compared to the follicular phase. Women wanted rewards significantly less when their levels of estradiol were high (p = .011) but the significant estradiol x reward type interaction showed that the negative relationship between estradiol levels and wanting ratings is attenuated for money compared to food rewards (p = .016). For men, there were no significant main effects of hormone levels on the task variables (all p > .05), but a significant interaction of

estradiol x reward type showed that in men, wanting ratings increase with rising levels of estradiol for money compared to food rewards (p = .044). Women indicated less wanting with higher 'feminine' ratings (p = .049). A significant interaction of 'feminine' x reward magnitude suggests a stronger association of higher wanting ratings for high rewards with higher 'feminine' ratings (p = .002). The significant 'feminine' x uncertainty term indicates that, with higher 'feminine' ratings, wanting ratings decrease for rewards in uncertain trials (p = .001). Significant interactions of 'masculine' x reward magnitude (p = .023) showed that the positive influence of a high reward on ratings of wanting was stronger if women reported higher 'masculine' ratings (p = .023). The significant term 'masculine' x reward type revealed that higher 'masculine' ratings in women are associated with reduced wanting ratings for money rewards (p = .006). For men, there were no significant main effects of 'feminine' and 'masculine' ratings on wanting (all p > .05). A significant term of 'feminine' x reward type revealed that the positive impact of money as a reward on wanting ratings was weaker for higher 'feminine' ratings (p = .008) and 'feminine' x difficulty showed that the negative effect of high difficulty trials on wanting was stronger when men had higher 'feminine' ratings (p = .032).

Exertion

Analyses did not show a significant sex difference in exertion (p = .061), but a significant effect of sex x reward magnitude showed that men indicate more exertion in response to high rewards compared to women (p = .011). All participants indicated they exerted more effort for high rewards (p < .001), money rewards (p < .001) and if task difficulty was medium compared to low (p = .029). All participant indicated they exerted more effort in uncertain trials compared to certain trials (p = .014). Women rated their exertion significantly lower in the periovulatory (p < .001) and luteal (p = .008) phases compared to the follicular phase. Women reported they exerted less effort with higher levels of estradiol (p < .001), but a significant interaction of estradiol x reward type showed that the positive effect of money on ratings of exertion was amplified with higher levels of estradiol (p = .009). Men also indicated less exertion for rewards with higher estradiol levels (p = .034). Women reported lower exertion with higher 'feminine' ratings (p = .016). A significant interaction of 'masculine' x reward type suggests that the effect of money rewards on exertion decreases when 'masculine' ratings are high (p < .001). A significant 'masculine' x difficulty term showed that the effect of medium difficult trials on exertion increases with higher 'masculine' ratings (p = .011). For men, exertion ratings were higher with higher 'feminine' ratings (p = .006). A significant term of 'feminine' x reward magnitude suggests that the effect of high rewards on exertion increases with higher 'feminine' ratings (p = .045). Significant interactions of 'feminine' x reward type and 'feminine' x difficulty indicated that the effect of money rewards (p = .003) and high difficulty (p = .016) decrease with higher 'feminine' ratings.

S11. Sex Hormone Levels as Measured in Study Part 1 and 2

Table S11. ***Cross-sectional hormone levels as measured during the initial session T0 and longitudinal data throughout sessions T1-T4 (N = 94)***

	Estradiol (pmol/L)			Progesterone (nmol/L)			Testosterone (nmol/L)		
	Range	*Mdn*	*IQR*	*Range*	*Mdn*	*IQR*	*Range*	*Mdn*	*IQR*
**T0: Cross-sectional**									
**Women** (n = 48)									
Follicular group days +1 to +9 (n = 18)	79-525	151	73	0.9-16.4	2.2	1.0	0.5-1.9	1.0	0.3
Periovulatory group days +10 to -11 (n = 12)	147-1104	320	420	1.3-2.9	2.1	1.2	0.7-1.4	1.0	0.3
Luteal group days -12 to -1 (n = 18)	119-1093	386	324	2.4-46.8	14.8	19.7	0.6-1.8	1.1	0.6
**Men** (n = 46)	51-312	132	51	0.5-3.4	1.9	1.1	7.9-43.9	18.0	7.3
**T1-T4: Longitudinal**									
**Women** (n = 48)									
Follicular phase days +3 to +13	68-1787	202	156	0.5-4.1	1.9	1.1	0.5-1.7	1.0	0.3
Periovulatory phase days +14 to -12	147-1302	440	323	0.9-31.6	3.1	2.5	0.5-1.7	1.1	0.4
Luteal phase days -11 to -4	11-1102	490	394	1.8-70.2	21.8	31.6	0.5-1.7	0.9	0.4
Perimenstrual phase days -3 to +2	76-695	191	102	1.3-45.7	3.9	5.0	0.4-1.6	0.8	0.4
**Men** (n = 46)									
Morning (n = 22) 7 am to 12 pm	68-270	126	53	0.5-4.5	2.3	1.5	4.2-29	17.3	8.95
Afternoon (n = 24) 12 pm to 6 pm	54-226	115	45	0.8-3.4	2.0	0.9	3.2-29.5	15.8	7.92

Note. Range, median (Mdn) and inter quartile range (IQR) for levels of estradiol, progesterone and testosterone. For the longitudinal data, for men, measures obtained in the morning and the afternoon are displayed.

S12. Estimates of Mixed-Effects Models for Hormone Fluctuations (Study Part 2)

Table S12. ***Estimates of mixed-effects models for hormone fluctuations between cycle phases for study part 2 (T1-T4)***

	**Estradiol**				**Progesterone**				**Testosterone**			
	β	*SE*	*t*	*p*	*β*	*SE*	*t*	*p*	*β*	*SE*	*t*	*p*
**Women**												
Follicular – peri-ovulatory	-0.63	0.1	-5.03	**<.001**	-0.58	0.2	-3.68	**.002**	-0.10	0.1	-2.52	.062
Peri-ovulatory – luteal	-0.05	0.1	-0.37	.983	-1.63	0.2	-9.80	**<.001**	0.17	0.1	3.89	**<.001**
Luteal – peri-menstrual	0.82	0.1	6.18	**<.001**	1.17	0.2	7.04	**<.001**	0.13	0.1	2.88	**.024**
	F		*df*	*p*	*F*		*df*	*p*	*F*		*df*	*p*
**Men**												
Morning – afternoon	3.98		1, 181	**.048**	3.35		1, 181	.069	2.09		1, 181	.150

Note. For women, mixed-effects models were fitted with estradiol, progesterone and testosterone (log-transformed) as outcomes, cycle phase (follicular = 0, periovulatory = 1, luteal = 2, perimenstrual = 3) as predictor and participant ID as the random effect. Session was used to control for order effects. For men, ANOVA were performed.

S13. Detailed Results of Motivational Behaviour Across the Menstrual Cycle (Figure)

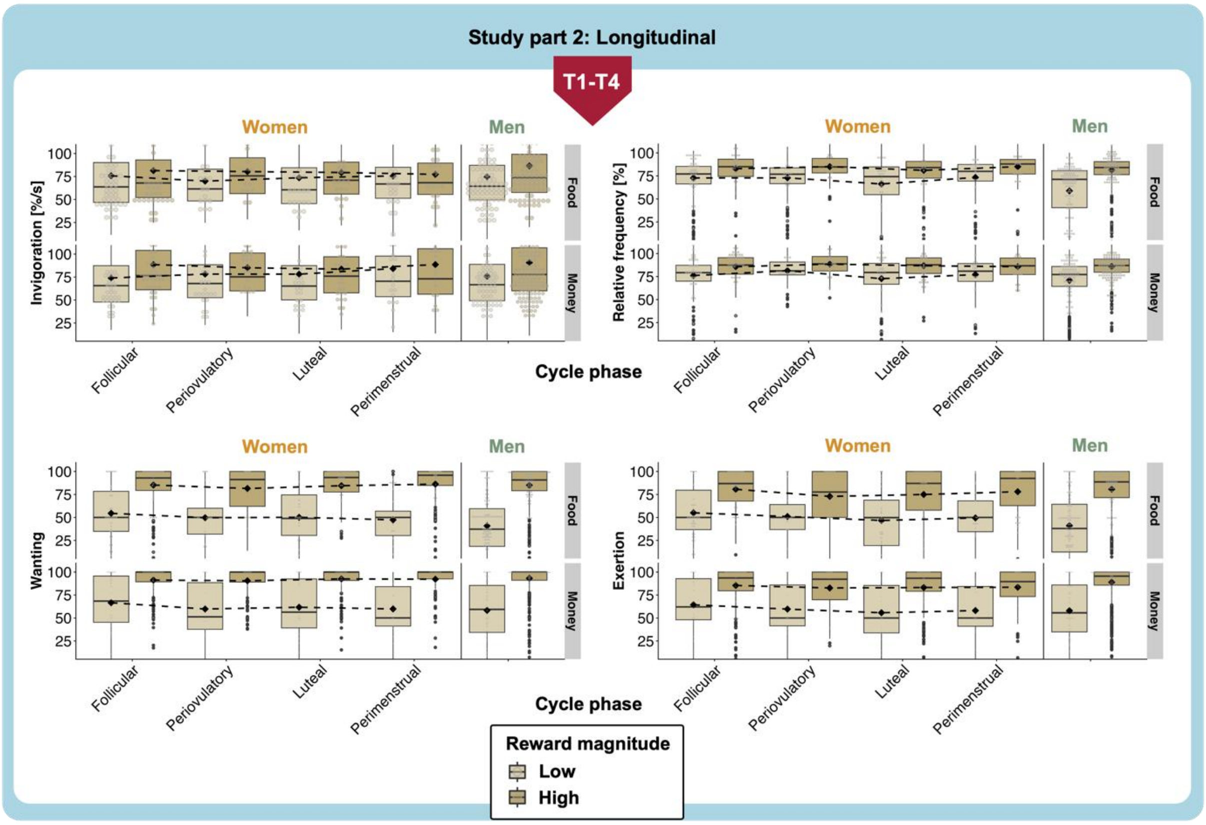

Figure S13. **Study part 2: Within-subjects analyses of cycle phases (T1-T4).**
Trial-based data showing that, when women were in the periovulatory phase, their ratings of wanting and exertion for the reward at stake were significantly decreased (main effect of cycle phase, both *p* < .001). In the luteal phase, women significantly decreased their effort maintenance and exertion ratings (main effect of cycle phase, *p* = .041 for effort maintenance and *p* = .008 for exertion), however, this effect was diminished for high rewards (interaction cycle phase x reward magnitude, *p* = .045) and money rewards (interaction cycle phase x reward type, *p* = .012).Women's wanting ratings were moderated by reward magnitude when they were in the perimenstrual phase, with higher wanting for high rewards (interaction cycle phase x reward magnitude, *p* = .002).

S14. Associations of Sex Hormone Levels with Motivational Behaviour (Figure)

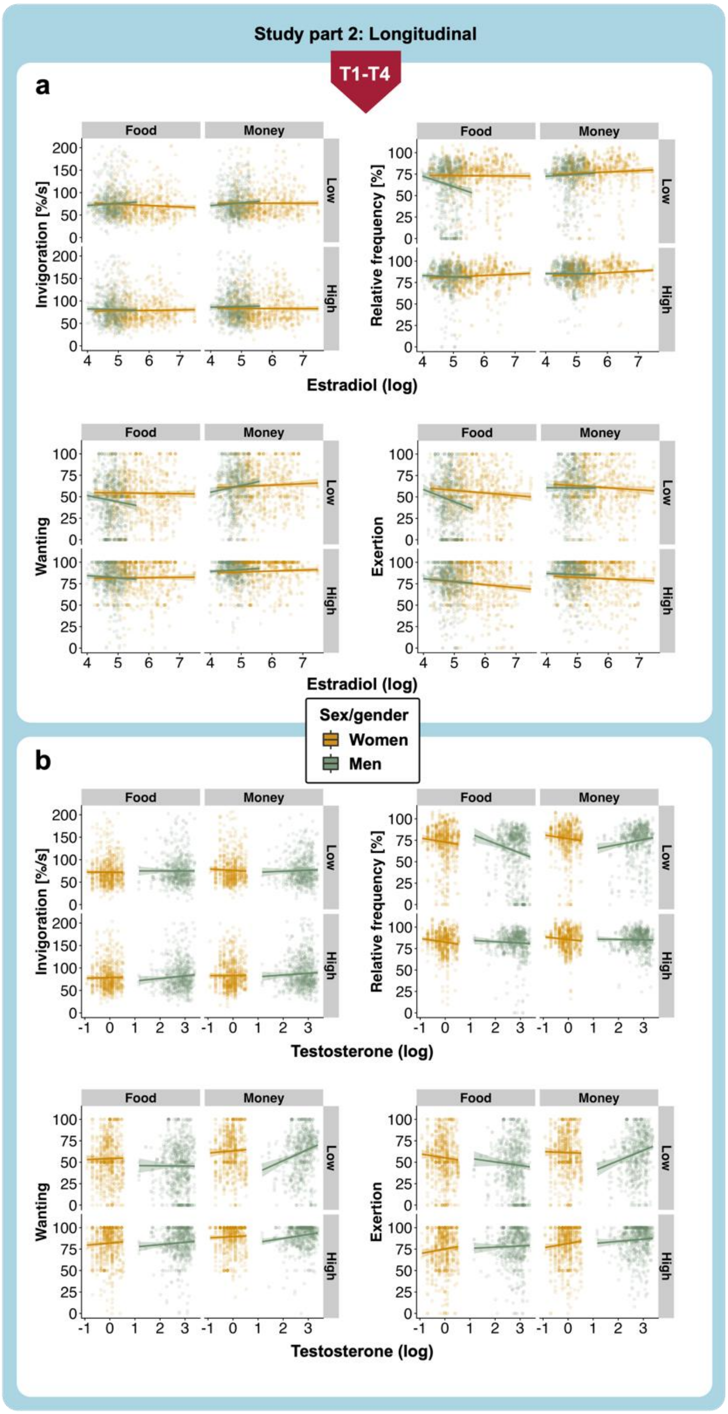

Figure S14. **Study part 2: Within-subjects analyses of hormones (T1-T4).**
Trial-based data showing that, for women, higher levels of estradiol are associated with a significant decrease in all measures of motivational behaviour, i.e. invigoration (main effect of estradiol, $p = .030$), effort maintenance (main effect of estradiol, $p = .017$), wanting (main effect of estradiol, $p = .011$) and exertion (main effect of estradiol, $p < .001$). For men, exertion ratings decreased overall with higher levels of estradiol (main effect of estradiol, $p = .034$).

G1. Description of the Gender-Related Attributes Survey (GERAS)

To assess gender expression, we employed a self-report measure assessing gender role identity (Gender-Related Attributes Survey, GERAS)[10]. The GERAS is based on positive and negative attributes that are typically associated with female or male gender in middle European cultures[30]. It comprises 50 items representing three subscales, where half of the items describe feminine attributes, and the other half describe masculine attributes. On the subscale 'Personality' (20 items), participants indicate how often certain traits apply to them on a 7-point Likert scale ranging from 1 (*never*) to 7 (*always*). For 'Cognition' (14 items), subjects rate how well they are able to solve certain problems and in the 'Activities & Interests' subscale (16 items), they indicate how much they like certain activities on a 7-point Likert scale from 1 (*not at all*) to 7 (*very*). Masculinity and femininity scores are calculated for each of the three subscales separately and for the inventory as a whole (average of individual ratings for feminine and masculine items).

In the current study, women and men differed significantly with regard to their gender role identity: On average, women indicated significantly higher overall femininity scores ($p < .001$) and men obtained higher masculinity scores ($p < .001$), see Table 1 in the main manuscript.

G2. Gender State Ratings

At the beginning of each session, participants completed a set of state ratings. To assess gender expression, participants indicated how ‘feminine’ and ‘masculine’ they currently felt. Questions were implemented as Visual Analogue Scales (VAS) ranging from 0 (*not at all*) to 100 (*very much*) on a computer screen and participants could move a cursor along the scale by use of an Xbox controller’s joystick and select their answer by pressing a button.

G3. Statistical Analyses including Gender Expression

Gender state ratings ('feminine' and 'masculine') completed in the beginning of each session were analysed separately for women and men. Independent samples *t*-tests (study part 1) and mixed-effects models (part 2) were performed to investigate significant differences in how 'feminine' and 'masculine' women and men indicated they felt. In study part 1 (T0), an ANOVA was used to check if 'feminine' and 'masculine' ratings differed significantly between cycle phase groups. For longitudinal data in study part 2 (T1-T4), mixed-effects models were fitted, with 'feminine' and 'masculine' ratings as outcomes and sex/gender and cycle phase as predictors, session to control for order effects and participant ID as the random effect.

To investigate whether gender expression influences motivational behaviour and subjective ratings of wanting and exertion differentially from sex/gender identity, we included 'feminine' and 'masculine' gender state ratings as predictors in the mixed effects models described in the main manuscript.

G4. Results Section on Gender Expression

Gender Expression Affects Motivation Differently for Women and Men

Study Part 1: Between Subjects (T0)

Women and men indicated significant differences in their ratings of how 'feminine' and how 'masculine' they felt in the beginning of the initial session. Women ($M_{feminine}$ = 73.14, $SD_{feminine}$ = 21.28) obtained higher ratings on the 'feminine' VAS compared to men ($M_{feminine}$ = 28.76, $SD_{feminine}$ = 20.03), t(91.97) = 10.42, p < .001, and men ($M_{masculine}$ = 60.86, $SD_{masculine}$ = 14.73) had higher ratings on the 'masculine' VAS than women ($M_{masculine}$ = 18.67, $SD_{masculine}$ = 19.60), t(87.12) = -11.83, p < .001. There were no significant differences in ratings dependent on cycle phase group or hormone levels, all p > .05, see Figure G5 a and c.

Gender expression influenced motivational behaviour in women but not in men (all p > .05 for men). For objective measures in women, higher 'feminine' ratings were associated with higher effort maintenance (b = 0.37, p = .009, Cohen's d = 0.04) and less pronounced increases in effort maintenance for high rewards (b = -0.39, p = .002 , Cohen's d = -0.04). This suggests that women who perceived themselves as more feminine tended to raise their effort more for low rewards. Higher 'masculine' ratings decreased the positive effect of high rewards on invigoration (b = -0.25, p = .025, Cohen's d = -0.01), meaning that women who felt more masculine did not increase their initial effort as much in response to higher rewards.

Subjectively, women with higher 'feminine' scores reported higher wanting (b = 0.43, p = .009, Cohen's d = 0.03) and higher exertion (b = 0.40, p = .023, Cohen's d = 0.03), indicating that, in women, a more feminine self-perception was linked to a higher desire for rewards and greater willingness to work for them. Both higher 'feminine' and higher 'masculine' ratings reduced the positive effects of reward magnitude on wanting (b = -0.51, p = .002, Cohen's d = -0.03 for 'feminine' and b = -0.35, p = .043, Cohen's d = -0.02 for 'masculine') and exertion (b = -0.56, p < .001, Cohen's d = -0.04 for 'feminine' and b = -0.39, p = .015, Cohen's d = -0.02 for 'masculine'), see Figure G6 a and c. This shows that as women's gender expression became more pronounced, their motivational response to higher rewards diminished, resulting in reduced subjective sensitivity to high incentives.

Study Part 2: Within Subjects (T1-T4)

There was an overall sex/gender effect on ratings of 'feminine' and 'masculine' for women and men across T1-T4. Women ($M_{feminine}$ = 70.28, $SD_{feminine}$ = 23.51) obtained higher ratings on the 'feminine' VAS compared to men ($M_{feminine}$ = 25.34, $SD_{feminine}$ = 20.67), b = -44.86, p < .001, Cohen's d = 2.03, and men ($M_{masculine}$ = 69.45, $SD_{masculine}$ = 16.42) obtained higher ratings on the 'masculine' VAS than women ($M_{masculine}$ = 22.49, $SD_{masculine}$ = 22.19), b = 46.49, p < .001, Cohen's d = -2.41. Similar to the cross-sectional data, there were no significant differences in ratings in the course of the menstrual cycle or dependent on fluctuating hormone levels (T1-T4), all p > .05, see Figure G5 b and d.

Both women and men exhibited significantly lower objective effort maintenance with higher 'feminine' ratings, i.e., they were less consistent in sustaining their effort and more sensitive to effort costs when they rated themselves as more feminine (b = -0.10, p < .001, Cohen's d = -0.01 for women, b = -0.14, p = .002, Cohen's d = -0.01 for men). However, these effects were less pronounced for high rewards (b = 0.08, p = .005, Cohen's d = 0.01 for women; b = 0.15, p < .001, Cohen's d = 0.01 for men), indicating that the motivational boost from larger rewards counteracted the negative effect of femininity. For women, higher 'masculine' ratings were associated with lower effort maintenance in response to monetary rewards (b = -0.11, p < .001, Cohen's d = -0.01). For men, 'masculine' ratings showed a positive association with increased invigoration (b = 0.38, p = .008, Cohen's d = 0.01) and decreased effort maintenance (b = -0.15, p = .002, Cohen's d = -0.01). However, the latter negative impact was reduced when high rewards were at stake (b = 0.14, p = .004, Cohen's d = 0.01), showing that larger incentives mitigated this decline in sustained effort.

Regarding subjective ratings, women reported significantly lower wanting (b = -0.08, p = .049, Cohen's d = -0.01) and exertion (b = -0.12, p = .016, Cohen's d = -0.01) when 'feminine' ratings were high. The negative effect of 'feminine' ratings on wanting was diminished for high rewards (b = 0.12, p = .002, Cohen's d = 0.01). Men's exertion ratings were higher with increased 'feminine' ratings (b = 0.20, p = .006, Cohen's d = 0.01), particularly for high rewards (b = 0.13, p = .045, Cohen's d = 0.01), while this effect was attenuated for monetary rewards (b = -0.19, p = .003, Cohen's d = -0.01). Further, for men, higher 'feminine' ratings decreased the positive impact of monetary rewards on wanting (b = -0.16, p = .008, Cohen's d = -0.01), indicating that more pronounced feminine gender expression rendered men less motivated by monetary rewards. For women, higher 'masculine' ratings were associated with increased wanting of high rewards (b = 0.09, p = .023, Cohen's d = 0.01), but lower wanting (b = -0.10, p = .006, Cohen's d = -0.01) and exertion ratings (b = -0.17, p < .001, Cohen's d = -0.01) in response to monetary rewards, indicating a complex relationship of gender expression and motivational behaviour with distinct patterns for women and men, see Figure G6 b and d.

G5. Results of Gender State Ratings (Figure)

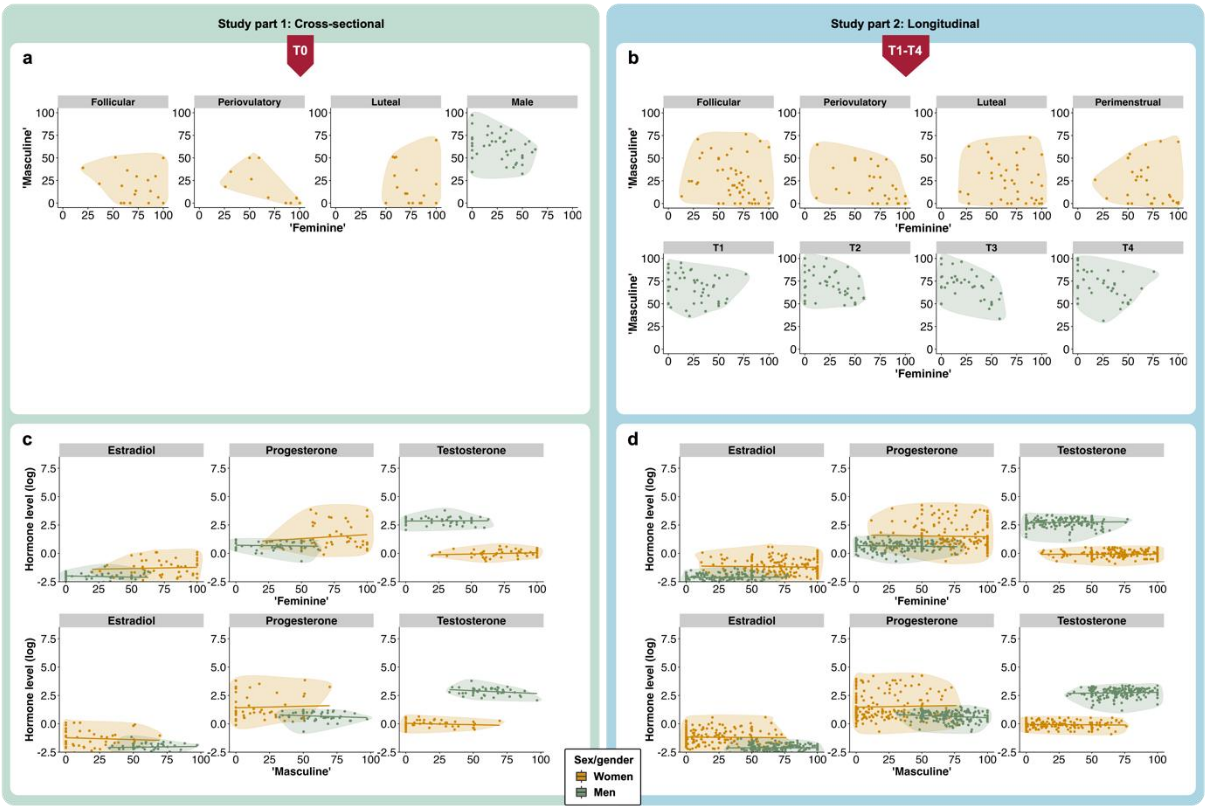

***Figure G5*. Gender state ratings ('feminine' and 'masculine') of women (orange) and men (green).**

(**a**) separately for each cycle phase group (follicular, periovulatory and luteal group) at T0, (**b**) across the menstrual cycle (follicular, periovulatory, luteal and perimenstrual phase) or across sessions for T1-T4, (**c**) dependent on sex hormone levels of estradiol, progesterone and testosterone as measured at T0 (log values, between-subjects analyses) and (**d**) dependent on sex hormone levels during T1-T4 (log values, within-subjects analyses).

G6. Associations of Gender State Ratings and Motivational Behaviour (Figure)

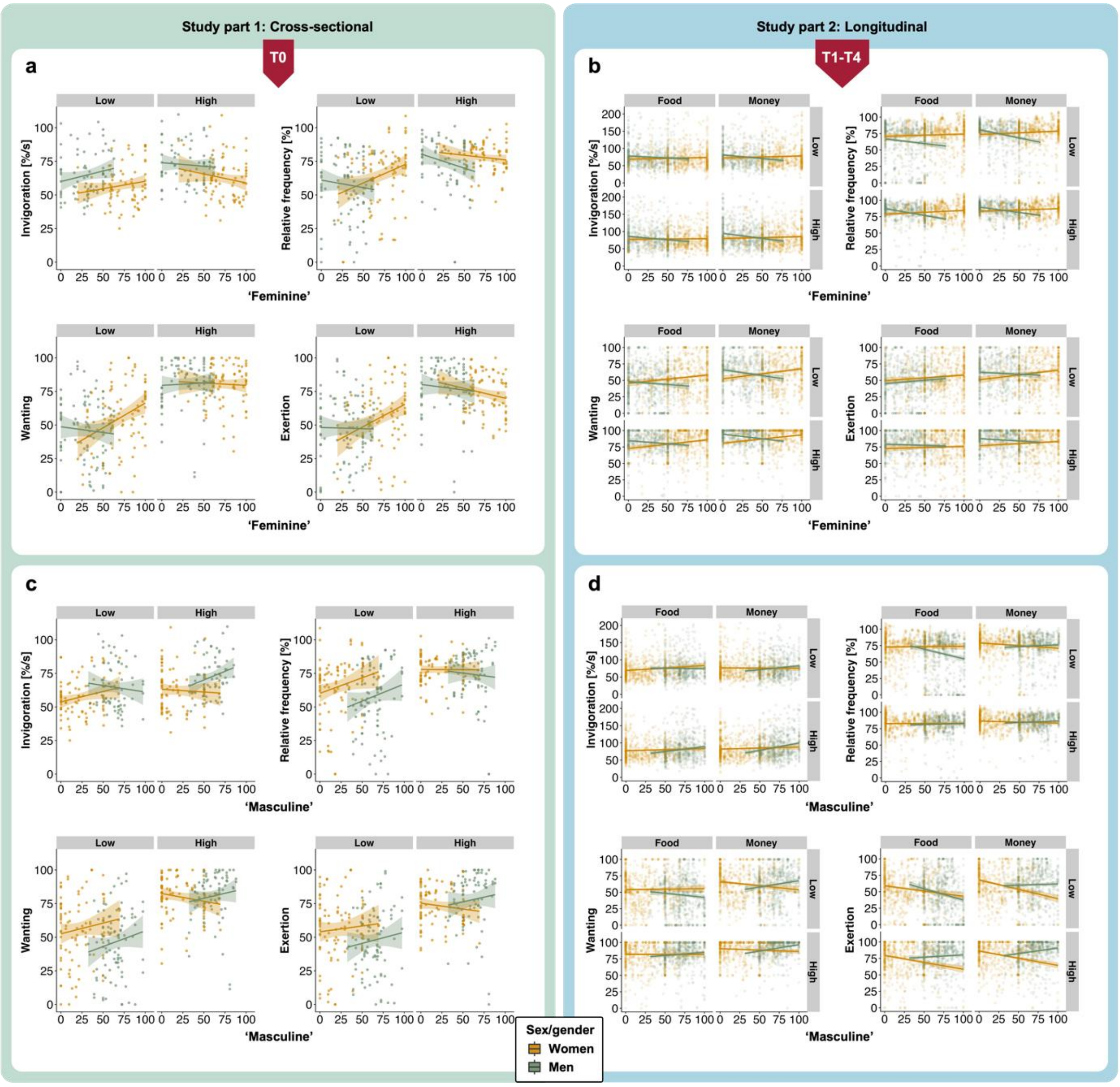

***Figure G6*. Associations of gender state ratings of women (orange) and men (green) with motivational behaviour.**

(**a**) Motivational behaviour dependent on 'feminine' ratings at T0 and (**b**) T1-T4, and (**c**) motivational behaviour dependent on 'masculine' ratings at T0 and (**d**) T1-T4.

G7. Discussion of Findings on Gender Expression

Gender Expression Influences Effort Allocation

Gender expression ratings influenced motivation differently in women and men across both study parts. In part 1, men's motivational behaviour was largely unaffected by gender expression, while women who rated themselves as more 'feminine' showed more sustained effort, greater desire for rewards, and higher willingness to work for rewards, although these effects diminished for high rewards. Higher 'masculine' ratings in women reduced the effect of high incentives on initial effort and decreased subjective sensitivity to high rewards. In part 2, both women and men were more sensitive to effort costs with higher 'feminine' ratings, sustaining effort less for rewards. Women's subjective wanting and exertion decreased with higher 'feminine' ratings while men's exertion ratings increased. These effects were less pronounced for high rewards and more for monetary rewards. Higher 'masculine' ratings increased initial effort in men but decreased effort maintenance, whereas for women, they decreased effort maintenance, wanting and exertion for monetary rewards, but increased wanting for high rewards.

Although gender-related patterns varied across study parts, they suggest that feminine traits may promote consistent, sustained effort across different motivational measures, possibly reflecting a focus on meeting task demands. Masculine traits might encourage a more opportunistic approach, boosting initial motivation but reducing sustained effort, with nuanced variations depending on reward magnitude and type.

The terms 'feminine' and 'masculine' in the context of this study refer to gender expression as assessed through self-report measures, which capture individuals' perception of how much they embody traits associated with femininity or masculinity. These self-perceived traits are shaped by experience, social desirability and societal expectations[31] of how women and men should behave[32,33]. Gradational scales of femininity and masculinity as used in the current study have been shown to reflect gender diversity well and constitute a comprehensive measure of self-identification that allow for an assessment of gender identification along dimensions[34]. Femininity and masculinity scales do not represent a fixed sense of gender but how a person perceives their position within broader gender constructs[35]. Social gender roles are internalised early in life and are reflected in individuals' personality traits, whereby women have been described as, for instance, more conscientious and men as more risk-taking[36]. Feminine traits are typically associated with qualities such as agreeableness and empathy, whereas masculine traits are traditionally linked to assertiveness and competitiveness[37,38]. A feminine, socially desirable approach to completing the reward task might be optimal task performance, including conscientious adherence to task instructions. A masculine strategy would be more competitive, focusing on maximising reward gains and conserving energy in trials with less important rewards in order to increase effort for more interesting rewards. This notion is consistent with previous sex/gender differences found in a gambling task, where men demonstrated a greater focus on long-term payoffs compared to women[39].

The discrepancies in findings from study part 1 and 2 highlight the variability in gender-related influences on motivational behaviour, which may reflect different contexts and timings of sessions within the study parts, as well as overall variability in gender expression. Our findings reveal a complex interplay between gender expression and motivational dynamics, suggesting that gender, beyond biological sex, significantly influences how individuals engage in and perceive effort-related tasks, and may also influence motivational behaviour more broadly. These small but nuanced effects highlight the importance of considering gender alongside biological factors in understanding motivational behaviour and performance in reward-based tasks. Using a biopsychosocial approach, we further sought to disentangle the associations of sex and gender with specific motivational behaviours and also investigated whether menstrual cycle phase and fluctuations in sex hormone levels modulate these effects[40].

The present study has several limitations that may influence future research. Gender expression did not vary significantly over time or across the menstrual cycle. To gain a more nuanced understanding of sex/gender differences and to avoid the scientific simplification of sex[41] and gender[34], the construct of gender should be assessed in more detail considering its multidimensional nature[42]. Causal analyses of the reciprocal interactions between sex and gender remain difficult, as some sex- and gender-related processes may moderate or reinforce each other, or may be simultaneously influenced by additional factors, such as specific phases of the menstrual cycle or sex hormone fluctuations. In addition, the study sample consisted predominantly of Caucasian participants with high levels of education, which limits the generalisability of our findings. A recent systematic review[43] showed that countries with better living conditions have larger sex/gender differences in personality and cognitive functioning, supporting the 'resource hypothesis'[44], which suggests that in countries where basic material needs are met, individuals have more freedom to pursue sex- and gender-specific goals and aspirations. Future research should therefore include more diverse and representative samples to better understand the interplay between sex, gender and corresponding socioeconomic factors on motivational behaviour.

Gender represents an emerging field in behavioural neuroscience, where we explore a construct beyond the binary[45]. The significant influence of gender expression on motivational behaviour, particularly in women, highlights the importance of considering psychosocial factors alongside biological factors in understanding sex

and gender differences in motivation. Our findings may have implications for understanding sex and gender differences in motivational contexts, such as work performance, sport and therapeutic interventions. Future research should continue to explore these dynamics by incorporating not only biological, but also psychosocial factors and individual differences in reward sensitivity to further elucidate the underpinnings of motivational behaviour. The results of our study could also inform improved treatments for mental disorders characterised by deficits in reward learning, such as mood disorders.

Supplementary References

1. Neuser MP, Kräutlein F, Kühnel A, Teckentrup V, Svaldi J, Kroemer NB. Influenca: a gamified assessment of value-based decision-making for longitudinal studies. *bioRxiv*. Published online April 28, 2021:2021.04.27.441601. doi:10.1101/2021.04.27.441601
2. Guitart-Masip M, Huys QJM, Fuentemilla L, Dayan P, Duzel E, Dolan RJ. Go and no-go learning in reward and punishment: Interactions between affect and effect. *Neuroimage*. 2012;62(1):154-166. doi:10.1016/J.NEUROIMAGE.2012.04.024
3. Kühnel A, Teckentrup V, Neuser MP, et al. Stimulation of the vagus nerve reduces learning in a go/no-go reinforcement learning task. *European Neuropsychopharmacology*. 2020;35:17-29. doi:10.1016/J.EURONEURO.2020.03.023
4. Breyer B;, Bluemke M. Deutsche Version der Positive and Negative Affect Schedule PANAS (GESIS Panel). *20*. Published online 2016:20. doi:10.6102/ZIS242
5. Leiner DJ. SoSci Survey (Version 3.5.02). Published online 2024. Accessed March 13, 2024. https://www.soscisurvey.de
6. Steiner M, Macdougall M, Brown E. The premenstrual symptoms screening tool (PSST) for clinicians. *Arch Womens Ment Health*. 2003;6(3):203-209. doi:10.1007/S00737-003-0018-4/METRICS
7. Bohn MJ, Babor TF, Kranzler HR. The Alcohol Use Disorders Identification Test (AUDIT): validation of a screening instrument for use in medical settings. *http://dx.doi.org/1015288/jsa199556423*. 2015;56(4):423-432. doi:10.15288/JSA.1995.56.423
8. Heatherton ToddF, Kozlowski LynnT, Frecker RC, Fagerström KOO. The Fagerström Test for Nicotine Dependence: a revision of the Fagerstrom Tolerance Questionnaire. *Br J Addict*. 1991;86(9):1119-1127. doi:10.1111/J.1360-0443.1991.TB01879.X
9. Adamson SJ, Sellman JD. A prototype screening instrument for cannabis use disorder: The Cannabis Use Disorders Identification Test (CUDIT) in an alcohol-dependent clinical sample. *Drug Alcohol Rev*. 2003;22(3):309-315. doi:10.1080/0959523031000154454
10. Gruber FM, Distlberger E, Scherndl T, Ortner TM, Pletzer B. Psychometric Properties of the Multifaceted Gender-Related Attributes Survey (GERAS). *Eur J Psychol Assess*. 2019;36(4):612-623. doi:10.1027/1015-5759/A000528
11. Rosenberg M. Rosenberg Self-Esteem Scale. *PsycTESTS Dataset*. Published online September 12, 2011. doi:10.1037/T01038-000
12. Hautzinger M, Keller F, Kühner C. Beck-Depressions-Inventar: Revision. *Harcourt test services*. Published online 2006.
13. Stark R, Kagerer S, Walter B, Vaitl D, Klucken T, Wehrum-Osinsky S. Trait Sexual Motivation Questionnaire: Concept and Validation. *J Sex Med*. 2015;12(4):1080-1091. doi:10.1111/JSM.12843
14. Carver CS, White TL. Behavioral Inhibition, Behavioral Activation, and Affective Responses to Impending Reward and Punishment: The BIS/BAS Scales. *J Pers Soc Psychol*. 1994;67(2):319-333. doi:10.1037/0022-3514.67.2.319
15. Meule A, Reichenberger J, Blechert J. Development and preliminary validation of the Salzburg Stress Eating Scale. *Appetite*. 2018;120:442-448. doi:10.1016/J.APPET.2017.10.003
16. Meule A, Hermann T, Kübler A. A short version of the food cravings questionnaire-trait: The FCQ-T-reduced. *Front Psychol*. 2014;5(MAR):80398. doi:10.3389/FPSYG.2014.00190/BIBTEX
17. Pudel V, Westenhöfer J. *Fragebogen Zum Eßverhalten (FEV) - Handanweisung.* Verlag für Psychologie Dr. C. J. Hogrefe; 1989. Accessed April 27, 2025. https://reposit.haw-hamburg.de/handle/20.500.12738/5027
18. Lowe MR, Butryn ML, Didie ER, et al. The Power of Food Scale. A new measure of the psychological influence of the food environment. *Appetite*. 2009;53(1):114-118. doi:10.1016/J.APPET.2009.05.016
19. Fairburn C, Beglin S. Assessment of eating disorders: Interview or self-report questionnaire? *International journal of eating disorders*. 1994;16(4):363-370. Accessed April 27, 2025. https://onlinelibrary.wiley.com/doi/abs/10.1002/1098-108x(199412)16:4%3C363::aid-eat2260160405%3E3.0.co;2-%23?casa_token=lo1XJmX4BV8AAAAA:cJK3ODiNc3bz2uMqVEc-EcZGpUEdMxgLIvGgW4SfiDcpuj1FV_60jDZHQoDW_vM0aAQ5FvIoN9N4pxg
20. Gearhardt AN, Corbin WR, Brownell KD. Preliminary validation of the Yale Food Addiction Scale. *Appetite*. 2009;52(2):430-436. doi:10.1016/J.APPET.2008.12.003
21. Behr M, Becker M. SEE-Skalen zum Erleben von Emotionen (SEE). *Zeitschrift für Medizinische Psychologie*. 2006;141.
22. Craig CL, Marshall AL, Sjöström M, et al. International physical activity questionnaire: 12-country reliability and validity. *Med Sci Sports Exerc*. 2003;35(8):1381-1395. doi:10.1249/01.MSS.0000078924.61453.FB

23. Sockeel P, Dujardin K, Devos D, Denève C, Destée A, Defebvre L. The Lille apathy rating scale (LARS), a new instrument for detecting and quantifying apathy: validation in Parkinson's disease. *J Neurol Neurosurg Psychiatry*. 2006;77(5):579-584. doi:10.1136/JNNP.2005.075929
24. Liebowitz MR. Liebowitz social anxiety scale. *J Anxiety Disord*. Published online 1987. Accessed April 27, 2025. https://psycnet.apa.org/doiLanding?doi=10.1037%2Ft07671-000
25. Snaith RP, Hamilton M, Morley S, Humayan A, Hargreaves D, Trigwell P. A Scale for the Assessment of Hedonic Tone the Snaith–Hamilton Pleasure Scale. *The British Journal of Psychiatry*. 1995;167(1):99-103. doi:10.1192/BJP.167.1.99
26. Spielberger CD, Gonzalez-Reigosa F, Martinez-Urrutia A, Natalicio LFS, Natalicio DS. The State-Trait Anxiety Inventory. *Revista Interamericana de Psicología/Interamerican Journal of Psychology*. 1971;5(3 & 4):3-4. doi:10.30849/RIP/IJP.V5I3
27. Neuser MP, Teckentrup V, Kühnel A, Hallschmid M, Walter M, Kroemer NB. Vagus nerve stimulation boosts the drive to work for rewards. *Nat Commun*. 2020;11(1):1-11. doi:10.1038/s41467-020-17344-9
28. Schmalenberger KM, Tauseef HA, Barone JC, et al. How to study the menstrual cycle: Practical tools and recommendations. *Psychoneuroendocrinology*. 2021;123:104895. doi:10.1016/J.PSYNEUEN.2020.104895
29. Van De Pol M, Wright J. A simple method for distinguishing within- versus between-subject effects using mixed models. Published online 2008. doi:10.1016/j.anbehav.2008.11.006
30. Pletzer B, Steinbeisser J, Van Laak L, Harris TA. Beyond biological sex: Interactive effects of gender role and sex hormones on spatial abilities. *Front Neurosci*. 2019;13:675. doi:10.3389/FNINS.2019.00675/BIBTEX
31. Risman BJ. Gender as a social structure: Theory wrestling with activism. *Gender and Society*. 2004;18(4):429-450. doi:10.1177/0891243204265349
32. Eagly AH. *Sex Differences in Social Behavior: A Social-Role Interpretation*. Vol Psychology Press.; 2013. Accessed September 25, 2023. https://books.google.de/books?hl=en&lr=&id=sf8oxcuiSlsC&oi=fnd&pg=PR3&dq=Eagly,A.H.SexDifferencesinSocialBehavior:ASocial-RoleInterpretation(Erlbaum,1987).+&ots=5yHdyp0anR&sig=NN_1ud_mTefs_VZb6hzaZyUWBB0#v=onepage&q&f=false
33. Greenwald AG, Poehlman TA, Uhlmann EL, et al. Understanding and Using the Implicit Association Test: III. Meta-Analysis of Predictive Validity. *Association*. 2009;97(1):17-41. doi:10.1037/a0015575.supp
34. Magliozzi D, Saperstein A, Westbrook L. Scaling Up: Representing Gender Diversity in Survey Research. *Socius*. 2016;2. doi:10.1177/2378023116664352/ASSET/IMAGES/LARGE/10.1177_2378023116664352-FIG2.JPEG
35. Hart CG, Saperstein A, Magliozzi D, Westbrook L. Gender and health: Beyond binary categorical measurement. *J Health Soc Behav*. 2019;60(1):101-118.
36. MacGiolla E, Kajonius PJ. Sex differences in personality are larger in gender equal countries: Replicating and extending a surprising finding. *International Journal of Psychology*. 2019;54(6):705-711. doi:10.1002/IJOP.12529
37. Eagly AH, Mladinic A, Otto S. Are Women Evaluated More Favorably Than Men?: An Analysis of Attitudes, Beliefs, and Emotions. *Psychol Women Q*. 1991;15(2):203-216. doi:10.1111/j.1471-6402.1991.tb00792.x
38. Bem SL. Bem Sex Role Inventory. *J Pers Soc Psychol*. Published online September 12, 1981. doi:10.1037/t00748-000
39. van den Bos R, Homberg J, de Visser L. A critical review of sex differences in decision-making tasks: Focus on the Iowa Gambling Task. *Behavioural Brain Research*. 2013;238(1):95-108. doi:10.1016/J.BBR.2012.10.002
40. Wierenga LM, Ruigrok A, Aksnes ER, et al. Recommendations for a better understanding of sex and gender in neuroscience of mental health. *Biological Psychiatry Global Open Science*. Published online December 30, 2023:100283. doi:10.1016/J.BPSGOS.2023.100283
41. Velocci B. The history of sex research: Is "sex" a useful category? *Cell*. 2024;187(6):1343-1346. doi:10.1016/j.cell.2024.02.001
42. Egan SK, Perry DG. Gender identity: a multidimensional analysis with implications for psychosocial adjustment. *Dev Psychol*. 2001;37(4):451-463. doi:10.1037/0012-1649.37.4.451
43. Herlitz A, Hönig I, Hedebrant K, Asperholm M. A Systematic Review and New Analyses of the Gender-Equality Paradox. *Perspectives on Psychological Science*. Published online January 3, 2023. doi:10.1177/17456916231202685/ASSET/IMAGES/LARGE/10.1177_17456916231202685-FIG10.JPEG

44. Stoet G, Geary DC. The Gender-Equality Paradox in Science, Technology, Engineering, and Mathematics Education. *Psychol Sci*. 2018;29(4):581-593. doi:10.1177/0956797617741719/ASSET/IMAGES/LARGE/10.1177_0956797617741719-FIG5.JPEG
45. Joel D, Tarrasch R, Berman Z, Mukamel M, Ziv E. Queering gender: studying gender identity in 'normative' individuals. *Psychol Sex*. 2014;5(4):291-321. doi:10.1080/19419899.2013.830640
